# Impact of keto leucine and isoleucine on CHO cell central carbon metabolism and performance in fed-batch and steady-state perfusion

**DOI:** 10.3389/fbioe.2026.1708088

**Published:** 2026-02-17

**Authors:** Philipp Reifenberg, Daniel Benjamin, Maxime Le Mignon, Aline Zimmer

**Affiliations:** 1 Merck Life Science KGaA, Upstream R&D, Darmstadt, Germany; 2 Institute for Organic Chemistry and Biochemistry, Technische Universität Darmstadt, Darmstadt, Germany; 3 Metalytics, Inc., Cary, NC, United States

**Keywords:** bleed reduction, branched-chain keto acids, isoleucine, leucine, metabolic flux analysis, metabolomics, steady-state perfusion

## Abstract

The keto acids of isoleucine and leucine are bioavailable precursors of their branched-chain amino acids in Chinese hamster ovary (CHO) cells, which are used to produce biotherapeutics at industrial scale. In this study, the potential of branched-chain keto acids to improve product yield was evaluated in fed-batch and simulated steady-state perfusion. In fed-batch, combined or individual replacement of isoleucine and leucine at equimolar concentrations with their keto acids moderately increased (+6%) or maintained the cell-specific productivity *qP*, but this positive impact was counteracted by a reduction in cell growth up to −11%. Higher concentrations of keto acids substantially reduced cell growth (−42%) and *qP* (−25%). ^13^C-metabolic flux analysis during the growth phase of the fed-batch revealed that this detrimental effect may be associated with impaired glycolysis and TCA cycle activity, along with altered fluxes in anaplerotic reactions, ultimately leading to decreased ATP (−20%) and NADPH (−29%) generation. In steady-state perfusion, keto acid supplementation improved IgG yield up to 100% through (I) reduced bleed rates as a result of lower cell growth and (II) enhanced *qP*. Untargeted metabolite profiling demonstrated altered levels of various compounds, suggesting pathways that may be associated with the observed improvements. Overall, the findings of this study highlight the potential of novel media components, such as keto isoleucine and keto leucine, to improve yields and efficiency in biopharmaceutical production, thereby contributing to increased sustainability and lower manufacturing costs.

## 1 Introduction

Chinese hamster ovary (CHO) cells have served in the production of biotherapeutics for decades, yet efforts in research and development involving this cell expression system continue, relentlessly pushing the boundaries toward achieving higher yields at reduced costs. Biopharmaceutical manufacturers focus on improving the space-time yield, i.e., the total product yield per bioreactor volume and time, to enhance the overall efficiency of their production processes (Müller et al., 2022; Reger et al., 2023a,b; Lemire et al., 2025). Therefore, various aspects of the culture process, including cell lines, media formulations, and operating conditions, are permanently optimized to achieve higher cell densities, increased product titers, and improved product quality (Schulze et al., 2022; Xiang et al., 2024; Masuda et al., 2024; Gyorgypal et al., 2025).

The concentration of media and feeds is one approach to improve the space-time yield and sustainability in industrial processes using CHO cells (Schmidt et al., 2021). In terms of solubility limitations, the next critical media components after tyrosine and cysteine (in the form of cystine) are the branched-chain amino acids (BCAAs) leucine (Leu) and isoleucine (Ile). Replacement with their α-keto acids (KAs) keto leucine (KL) and isoleucine (KI) was demonstrated to be effective for feed concentration, and sufficient bioavailability was linked to transamination by branched-chain amino acid aminotransferase (BCAT). However, supplementation with high amounts of KI and KL in the feed indicated a detrimental impact on cell growth, viability, and productivity in fed-batch processes (Schmidt et al., 2020).

Despite the negative effects at toxic KI and KL concentrations, a balanced supply of KI and KL demonstrated moderately decreased cell growth while slightly increasing *qP*, i.e., the cell-specific productivity (Reifenberg et al., 2025). These characteristics are generally thought to be beneficial in steady-state perfusion, a state-of-the-art operation mode defined by the continuous inflow of fresh medium and outflow of spent medium while retaining the cells in the bioreactor (Maria et al., 2023). Steady-state operation aims to maintain a defined target cell concentration, requiring continuous removal of cells from the bioreactor, which results in a reduced harvest rate (Bielser et al., 2018, 2021; Schwarz et al., 2023). Despite recent technical advances, this commonly termed “bleed” is, to date, typically not processed further and is therefore considered waste, leading to considerable losses of the desired product (Wolf et al., 2019; Romann et al., 2023). Therefore, steady-state perfusion may profit from reduced cell growth, leading to lower bleed and consequently higher harvest rates. While cell cycle control through temperature shifts or the addition of sodium butyrate is state-of-the-art in CHO cultures (Avello et al., 2022), this study evaluates a novel approach using amino acid derivatives, KI and KL, to (I) reduce the bleed and (II) increase *qP*. A major advantage of this approach is the future perspective to concentrate the media, simplifying the solubilization and storage of media at a large scale and providing new opportunities to intensify processes.

In this study, ^13^C-metabolic flux analysis (MFA) was performed to investigate the impact of KI and KL on amino acid (AA) uptake and central carbon metabolism in a fed-batch process, replacing Ile and Leu with their KAs. MFA is a state-of-the-art technology that employs isotopically labeled substrates, such as ^13^C-labeled glucose, glutamine (Gln), or other AAs, as tracers to monitor the carbon flow through metabolic pathways of living cells (Antoniewicz, 2021; González-Hernández and Perré, 2024). The flux calculations are based on optimization techniques, fitting a fit-for-purpose metabolic model to experimental data by minimizing the difference between the observed reaction rates and those predicted by the model (Antoniewicz, 2021; de Falco et al., 2022; González-Hernández and Perré, 2024).

The quantification of intracellular fluxes in MFA differs significantly from the widely used untargeted metabolomics approach based on liquid chromatography (LC) coupled with high-resolution mass spectrometry (MS). Although this methodology lacks the detailed insights into pathway dynamics that MFA provides, it excels in comprehensively capturing the metabolic landscape and variations in metabolite abundance at a given time, without being constrained to a pre-defined set of reactions (Liang et al., 2021). Nevertheless, untargeted metabolomics faces challenges related to the complexity and diversity of metabolites present in biological samples. For instance, feature identification requires method-specific MS libraries, and relative quantification is complicated by varying ionization efficiency among different metabolites (Singh et al., 2024; Rakusanova and Cajka, 2024). Ultimately, MFA and untargeted metabolomics are thought to complement one another, providing a more holistic understanding of cellular metabolism (Jang et al., 2018; Liang et al., 2021).

In addition to ^13^C-MFA, which was performed to investigate the impact of KI and KL on the AA uptake and central carbon metabolism in fed-batch, the developability of the two branched-chain keto acids (BCKAs) as bleed reducers and *qP* enhancers in steady-state perfusion was evaluated in this study. This operation mode was simulated at small scale using two CHO cell lines, with KI and KL supplemented into commercially available cell culture medium. Subsequently, an untargeted metabolomics study was carried out to investigate the metabolic fate of the BCKAs and evaluate the hypothesis that the supplemented KAs induce changes in the metabolic landscape. The ultimate aim behind this approach was to identify pathways that may be involved in the intracellular events leading to the reduction in cell growth and the enhancement of productivity. Prospectively, the knowledge generated from the MFA and metabolomics studies might be crucial for maximizing the potential of these novel media components while minimizing the risk of undesired effects in industrial manufacturing settings. The ^13^C-MFA of the fed-batch process revealed that excessive BCKA supplementation through the feed strongly impaired glycolytic and TCA cycle activities while altering anaplerotic reactions, ultimately leading to reduced generation of adenosine triphosphate (ATP) and nicotinamide adenine dinucleotide phosphate (NADPH). While positive effects on IgG yield through increased *qP* were canceled out by reduced cell growth upon equimolar replacement (KI/KL) in fed-batch, the total product obtained in the harvest of the perfusion process increased substantially following BCKA supplementation as a result of reduced bleed and higher *qP*. Finally, the subsequent metabolomics study presented multiple impacted metabolites, suggesting pathways that may be further investigated to understand the intracellular events leading to the observed improvements.

## 2 Materials and methods

### 2.1 Chemicals, reagents and cell lines

The following reagents and standard compounds were obtained from Merck (Darmstadt, Germany): sodium chloride, ammonium formate, formic acid, acetonitrile, methanol, methyl-tert-butyl-ether, iodoacetamide, norvaline, O-benzylhydroxylamine, N-(3-dimethylaminopropyl)-N′-ethylcarbodiimide hydrochloride, L-glutamine, 3-methyl-2-oxopentanoic acid sodium salt, 4-methyl-2-oxopentanoic acid sodium salt, 3-methyl-2-oxobutyric acid sodium salt, ^13^C_5_-3-methyl-2-oxobutyric acid, ^13^C_6_,^15^N-L-leucine, 2-hydroxy-3-methylbutyric acid, 2-hydroxy-3-methylpentanoic acid, and 2-hydroxy-4-methylpentanoic acid. ^13^C_6_-4-methyl-2-oxopentanoic acid sodium salt was purchased from Cambridge Isotope Laboratories (Tewksbury, MA, United States). 1,2-^13^C_2_- and U-^13^C_6_-D-glucose, d_7_-2-hydroxy-3-methylbutyric acid, d_8_-3-methyl-2-oxopentanoic acid, d_3_-4-methyl-2-oxopentanoic acid, and d_3_-L-2-hydroxy-4-methylpentanoic acid were sourced from Toronto Research Chemicals (Toronto, ON, Canada). d_10_-2-Hydroxy-3-methylpentanoic acid (mixture of all four diastereomers) was synthesized internally from d_10_-isoleucine sourced from CDN Isotopes (Teddington, United Kingdom). Pierce™ Detergent-Compatible Bradford Assay Kit was purchased from Thermo Fisher Scientific (San Jose, CA, United States).

Chemically defined Cellvento® 4CHO, 4CHO-X medium, and ModiFeed Prime COMP (all three commercially available from Merck Life Science KGaA, Darmstadt, Germany) were used in this study to produce mAbs with a CHOK1 GS and CHO DG44 cell line. The sodium salts of KI and KL for cell culture were sourced in-house, and Ile and Leu were obtained from Merck Life Science KGaA (Darmstadt, Germany).

### 2.2 Metabolic flux analysis

#### 2.2.1 Fed-batch culture

Glutamine synthase (GS)-expressing CHOK1 cells were cultured in Cellvento® 4CHO medium without L-glutamine. Cellvento® ModiFeed Prime COMP was modified to be deficient in Ile and Leu, allowing for individual supplementation or replacement with KL and KI. Four conditions were tested by varying the Ile and Leu sources in Cellvento® ModiFeed Prime: (I) the positive control with Ile and Leu (“Ile/Leu”), (II) Ile and equimolar replacement of Leu by KL (“Ile/KL”), (III) equimolar replacement of both Ile and Leu by their BCKAs (“KI/KL”), and (IV) replacement of both Ile and Leu with their BCKAs with twice the original concentrations (“2KI/2KL”). Experimental conditions that include the ^13^C-tracers were performed in triplicate. One additional replicate per condition was run in parallel without ^13^C-tracer, serving as control. The fed-batch process was conducted in 50 mL spin tubes with vented caps (TPP, Trasadingen, Switzerland) at 37 °C, 5% CO_2_, 80% humidity, and a rotation speed of 320 rpm. Feeding schedules included the addition of 2.75%, 4.1%, 6%, 6%, and 2.75% (v/v) of the total volume on days 3, 5, 7, 10, and 13, respectively. Glucose concentrations were maintained by adding specific volumes of a 400 g/L glucose stock solution, targeting concentrations of 6 g/L during the week and up to 14 g/L before the weekend. Between days 4 and 7, cells were exposed to the ^13^C-tracer. Instead of supplementing exclusively native D-glucose up to 6 g/L, ^13^C-D-glucose molecules were introduced, resulting in 3, 1.5, and 1.5 g/L of native, 1,2-^13^C_2_-D-glucose, and U-^13^C_6_-D-glucose, respectively. This ratio of D-glucose species was maintained when supplementation was necessary within the exposure time. A Vi-CELL™ XR 2.04 cell counter (Beckman Coulter, Fullerton, CA, United States) was used to determine viable cell density (VCD) and viability. Cells were seeded with 2 × 10^5^ cells/mL in a 30 mL starting volume. The bioprocess analyzer Cedex Bio HT (Roche, Mannheim, Germany) was used for spent media analysis of glucose and IgG following centrifugation of the sample for 2.5 min at 2,000 rcf.

#### 2.2.2 Sampling

Aside from the daily sampling for VCD, viability, and spent media analysis, separate spent media samples and cell pellets were collected at 0, 64, 68 and 72 h after exposure to the ^13^C-tracers. Immediately after exposure (“T_0_”), 1 mL of cell suspension was centrifuged at 2,000 rcf for 2.5 min at 4 °C, and three 200 µL aliquots of the supernatant were stored at −20 °C until further use. Cell pellets from each individual replicate were obtained by quenching 1 mL of cell suspension with 4 mL of normal saline (0.9% NaCl, pre-chilled at 0.5 °C), gently mixing, and centrifuging at 1,000 rcf for 1 min at 4 °C. The supernatant was immediately removed by flipping and aspiration before the pellet was resuspended in 1 mL normal saline and transferred to a fresh microcentrifuge tube. Following centrifugation and aspiration of the supernatant, the remaining cell pellet was snap-frozen in liquid nitrogen and stored at −80 °C until further use. Quality-control pellets (n = 6) were taken on T_0_ from the unlabeled replicate of the positive control, according to the previously described quenching and washing process.

On T_64_, T_68_, and T_72_, spent media and cell pellets were collected similar to T_0_ sampling, but due to the increased VCDs, lower volumes of cell suspension (0.5 mL) were taken. Consequently, less normal saline (2 mL) was used for the quenching process.

After T_72_ sampling, three aliquots per biological replicate were taken for analysis of biomass composition. For each aliquot, 1 mL cell suspension was centrifuged at 1,000 rcf for 1 min at 4 °C, and the supernatant of one aliquot was transferred to a separate microcentrifuge tube. The other supernatants were removed by aspiration before the pellets of all aliquots were washed in 1 mL normal saline. The resulting cell pellets were snap-frozen in liquid nitrogen. The cell pellets and spent media were stored at −80 °C until further use.

#### 2.2.3 Sample analysis and flux calculations

In the spent media samples (*n* = 3), AAs, BCKAs, and branched-chain hydroxy acids (BCHAs) were quantified in-house using previously published methods (Reifenberg et al., 2025). In brief, AAs were quantified using the AccQ-Tag Derivatization Kit by Waters (Milford, MA, United States) and subsequent LC–UV analysis. Targeted LC-MS/MS analysis following O-benzylhydroxylamine derivatization was used to quantify BCKAs, while BCHAs were quantified from underivatized samples using a dedicated LC-MS method.

The collected cell pellets were provided to Metalytics, Inc. (Cary, NC, United States) for analysis of intracellular metabolites and biomass composition using multiple, proprietary methods. Combined with the cell culture and spent media data, Metalytics, Inc. performed the flux calculations using their in-house Core*MFA* software.

For the MFA study, the growth rate *µ* and the IgG production rate within the exposure period (T_0_ to T_72_) were calculated according to the following equations with the viable cell density [VCD] in 10^6^ cells/mL and the IgG concentration [IgG] in nmol/mL:

$$\text{Growth }\text{rate }µ\;\left[\frac{1}{h}\right]=\frac{\ln\left(\left[{\mathrm{VCD}}_{T72}\right]\right)-\ln\left(\left[{\mathrm{VCD}}_{T0}\right]\right)}{72h},$$

$$\text{IgG }\text{production }\text{rate }\left[\frac{\mathrm{nmol}}{10^{6}\frac{\mathrm{cells}}{h}}\right]=µ\times \frac{\left[{\mathrm{IgG}}_{T72}\right]-\left[{\mathrm{IgG}}_{T0}\right]}{{\left[\mathrm{VCD}\right.}_{T0}]\times \left(e^{µ\times 72h}-1\right)}.$$

### 2.3 Simulated steady-state perfusion

#### 2.3.1 Cell culture

CHOK1 cells, transfected with a plasmid containing the GS gene, were cultivated in Cellvento® 4CHO-X medium without supplementation of L-glutamine (Gln), while CHO DG44 cells, lacking the GS gene, were cultivated in Cellvento® 4CHO-X medium supplemented with 6 mM Gln. Glucose concentrations were increased to 10 g/L. For a short-term cultivation of 4 days, both cell lines were additionally cultured in their respective media supplemented with 1, 2.5, 5, 7.5, and 10 mM KI or KL.

Simulated perfusion processes were performed in 50 mL spin tubes with vented caps (TPP, Trasadingen, Switzerland) at 37 °C, 5% CO_2_, 80% humidity, and a rotation speed of 320 rpm. The starting cell density was 20 × 10^6^ cells/mL in a working volume of 20 mL. On a daily basis, the cell suspension was bled to readjust the culture to the target concentration of 20 × 10^6^ cells/mL, and the spent medium was exchanged for fresh medium to obtain a perfusion rate of 1. Cell counts, viability, and average viable cell diameter were measured using a Vi-CELL™ BLU cell counter (Beckman Coulter, Fullerton, CA, United States). Experimental conditions were performed with four replicates, unless stated otherwise. Spent media analysis, including IgG and pyruvate (Pyr), was performed using the bioprocess analyzer Cedex Bio HT (Roche, Mannheim, Germany) after centrifugation of the suspension for 1 min at 1,000 rcf. Cell pellets for the analysis of intracellular content were collected from the bleed and extracted according to a previously published procedure (Reifenberg et al., 2025). Both the spent media and intracellular content were subjected to AA analysis following the protocol reported in the same publication. Intracellular amino acid content was calculated as a percentage of the sum of all amino acids in the respective sample.

For the calculation of specific extracellular consumption/excretion rates, for example for *qP* and net AA consumption, the following equation was used, dividing the difference in the extracellular concentrations [X] between two days T_n_ and T_n-1_ by the time period [t] and the average of the cell concentration [VCD]:

$$\text{Consumption}/\text{excretion}\text{rate}=\frac{\left[X_{T_{n}}\right]-\left[X_{T_{n-1}}\right]}{t\times \frac{\left[{\text{VCD}}_{T_{n}}\right]+\left[{\text{VCD}}_{T_{n-1}}\right]}{2}}.$$

#### 2.3.2 Untargeted metabolomics

For relative quantitation of extra- or intracellular metabolites (*n* = 2), a previously established LC-MS method (Nguyen et al., 2023) with adjusted sample preparation and MS parameters was used on a UHPLC (Vanquish, Thermo Fisher Scientific, San Jose, CA, United States) coupled with an ESI-Q-ToF mass spectrometer (Impact II, Bruker Daltonics, Bremen, Germany). In brief, spent media samples were diluted tenfold in water, and dried intracellular content was reconstituted in 60 µL of water. A volume of 4 μL (extracellular) or 2 µL (intracellular) was injected onto an XSelect HSS T3 column (2.1 × 150 mm, 3.5 µm, Waters) in 99.9% buffer A (20 mM ammonium formate/0.1% formic acid) at 40 °C with a flow rate of 0.3 mL/min. 100% methanol (buffer B) was used for elution, applying the following linear gradient (min/% B): 0/0, 2/0, 4/20, 6/30, 8/80, 8.5/100, 9.5/100, 9.6/0, and 12/0.

MS data were acquired in positive and negative modes with capillary voltages at 4,500 and 3,500 V, respectively. Plate offset, nebulizer, and dry gas (250 °C) were set at 500 V, 1.4 bar, and 9.0 L/min, respectively. MS spectrum acquisition was performed in the range from *m/z* 50 or 100 to 1,000 with a scan rate of 2 Hz for metabolite quantification of intra- and extracellular samples, respectively. For metabolite identification, additional MS/MS data (*m/z* 50–1,000) were acquired from injections of individual samples with an MS scan rate of 12 Hz using a fixed MS-MS/MS cycle of 0.5 s and a dynamic exclusion list.

MetaboScape 2023b (Version 11.0.4, Bruker Daltonics, Bremen, Germany) was used for relative quantification of metabolites (peak area) using the T-ReX 3D workflow. The time frame for peak picking was set from 0.8 to 9.5 min with an intensity threshold of 500 counts and a minimum peak length of 8 spectra. QC samples were injected throughout the sequence and were used for “within batch-correction” to account for intensity drifts. Small molecules were annotated with different levels of confidence according to published recommendations (Sumner et al., 2007). In brief, tier 1 annotation required that the retention time (RT) and precursor *m/z* match within ±0.3 min and ±3.0 ppm, respectively. Moreover, MS/MS spectra needed to align with commercially available standards analyzed using the same method and instrument. In instances, where MS/MS data were unavailable, features were classified as tier 1’. Tier 2 annotation was assigned to features with matching precursor mass (±3.0 ppm) and MS/MS fragmentation pattern from spectral libraries (Bruker HMDB Metabolite and Bruker MetaboBASE personal libraries), while tier 3 level was achieved when the MS/MS fragmentation pattern of the putative structure was manually annotated. Tier 4 features were assigned with sum formulas with matching precursor mass (±5.0 ppm) and isotopic pattern (mSigma < 100). Finally, features only identified by a unique RT and precursor mass, which could not be annotated with a tentative structure or sum formula, were reported as tier 5.

### 2.4 Statistical analysis

Fed-batch and perfusion parameters are expressed as the mean ± standard error of three and four biological replicates, respectively, unless stated otherwise. Graphs were created using GraphPad Prism 10 software (Version 10.2.1, GraphPad Software Inc., San Diego, CA, United States). This software program was also used to carry out Mann–Whitney tests with individual fed-batch conditions (*p* < 0.05). For the untargeted metabolomics study (*n* = 2), R Studio (R version 4.1.2 (2021-11-01), Posit Software, PBC, Boston, MA) was used to calculate the area under the curve (AUC) of the individual features and to normalize the values of intracellular samples to the protein content of the extracted pellets (determined using Bradford assay). Normalization of values in intracellular samples was performed by dividing the peak area by the protein concentration. Furthermore, R Studio was applied to perform partial least squares (PLS) regression (Pareto-scaled; five components; leave-one-out validation) for each combination of (I) cell line (CHOK1 GS/CHO DG44), (II) supplemented keto acid (KI/KL), (III) MS source polarity (negative/positive), and (IV) sample type (extra-/intracellular). The *pls* (Hovde Liland et al., 2024) and *tidyverse* (Wickham et al., 2019) packages were used within RStudio.

## 3 Results

### 3.1 Metabolic flux analysis during fed-batch cultivation

#### 3.1.1 Fed-batch results distinguish two phenotypes upon BCKA supplementation

The impact of substituting Ile and Leu with their keto acid sodium salts, KI and KL, in Cellvento® ModiFeed Prime was investigated using a CHOK1 GS cell line in fed-batch culture. This study aimed to evaluate the BCKAs’ influence on central carbon metabolism during the growth phase via MFA. This technology was chosen due to its capability of computing intracellular reaction rates based on external rates and isotopic labeling within the explored pathways. For the cell culture experiment, the original feed formulation was depleted in Ile and Leu and subsequently supplemented with different amounts and combinations of Ile, Leu, KL, and racemic KI. The positive control was re-supplemented with Ile and Leu (“Ile/Leu”). Three additional feeds were prepared, comprising (I) Ile and KL, replacing Leu at equimolar concentration (“Ile/KL”), or substituting both Ile and Leu by KI and KL at (II) equimolar (“KI/KL”) or (III) two-fold concentrations (“2KI/2KL”).

**FIGURE 1:**
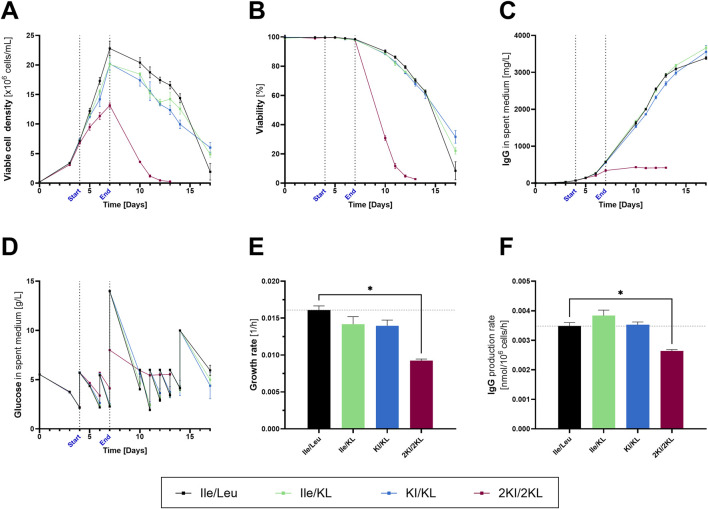
Viable cell density, viability, titer, glucose, growth rate, and IgG production rate obtained in fed-batch using CHOK1 GS cells with different combinations of branched-chain amino and keto acids in the feed formulation (mean ± SD, *n* = 4). Vertical, dotted lines indicate start (day 4) and end (day 7) of ^13^C-exposure through the addition of labeled glucose. **(A)** Viable cell density in 10^6^ cells/mL. **(B)** Viability in percent measured using the Vi-CELL XR. **(C)** IgG concentration in mg/L. **(D)** Glucose concentration in g/L measured using the Cedex Bio HT. **(E)** Growth rate between days 4 and 7 in h^−1^. **(F)** IgG productivity between days 4 and 7 in nmol/10^6^ cells/h. Mann–Whitney tests were performed **(E)** and **(F)** to verify statistically significant differences between Ile/Leu and 2KI/2KL (*p < 0.05).

The cell culture data (Figure 1) indicate two distinct phenotypes in terms of the BCKAs’ impact on parameters, such as cell growth, viability, and productivity. Following equimolar replacement of both or individual Ile and Leu by their KA, cell growth was moderately decreased (Ile/KL: −8%, KI/KL: −11%), and IgG titers remained similar. In clear contrast, peak VCD and IgG productivity were significantly reduced in the 2KI/2KL condition, followed by rapidly decreased viability from day 7 onward. The sudden decrease in viability was ascribed to reaching cytotoxic concentrations of KI (LD_50_ 17.9 mM) and KL (LD_50_ 14.6 mM) following feed addition after sampling on day 7 (Reifenberg et al., 2025). The presence of non-viable cells might have skewed the subsequent flux calculations. To ensure a fair comparison between all conditions, exposure to the ^13^C-tracer was restricted to the growth phase spanning day 4–7. A mixture of tracers 1,2-^13^C_2_- and ^13^C_6_-glucose was introduced on day 4 and 6, each compound aiming to comprise 25% of the total glucose pool. This combination enables isotope labeling of metabolites within glycolysis, the pentose phosphate pathway (PPP), the tricarboxylic acid (TCA) cycle, and closely related pathways.

The metabolic network of the MFA study included transport processes of nutrients and metabolic by-products as well as glycolysis, the pentose phosphate pathway, the TCA cycle including anaplerotic reactions, and AA metabolic reactions (Supplementary Table 1). In order to increase the accuracy of the model, reactions specific to the exogenous supplementation of BCKAs were added: (I) the BCAT-mediated transamination involving the BCKAs and BCAAs (rather than the natural substrate L-glutamate (Glu)) and the amination of (3R)-KI forming the non-proteogenic Allo-Ile and (II) the reduction of BCKAs leading to the extracellular accumulation of BCHAs (Schmidt et al., 2020; Reifenberg et al., 2025).

#### 3.1.2 High concentrations of BCKAs alter the uptake of multiple amino acids

**FIGURE 2:**
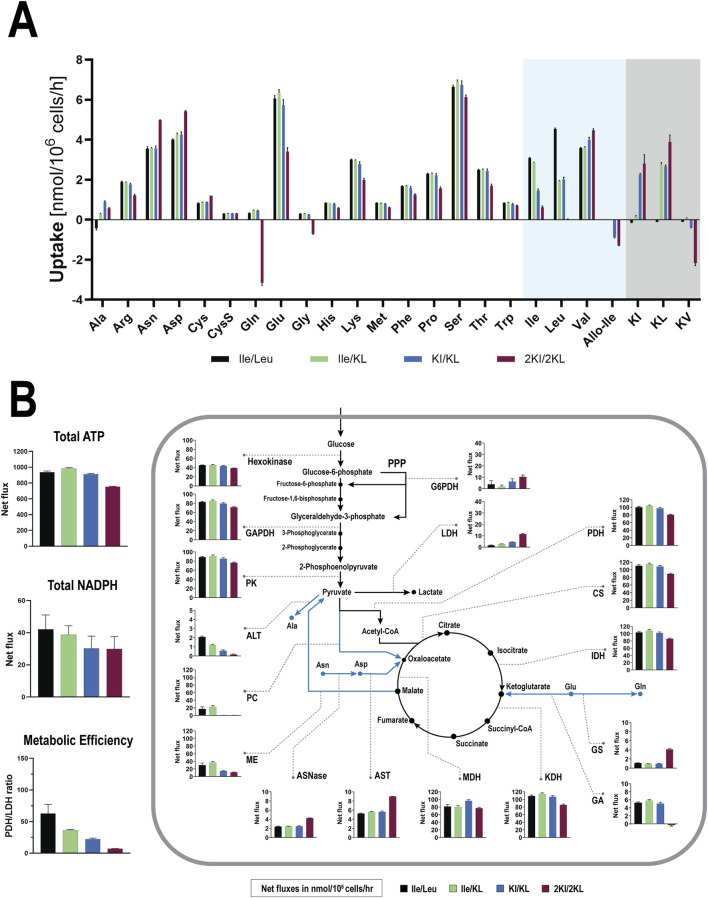
CHOK1 GS cells were examined using metabolic flux analysis during the growth phase of a fed-batch upon substitution of Ile and Leu by their corresponding branched-chain keto acids. **(A)** Amino acid and branched-chain keto acid uptake rates in nmol/10^6^ cells/h were calculated from extracellular concentrations (mean ± SD, *n* = 3). Negative values indicate net efflux. Branched-chain amino and keto acids are highlighted with blue and gray shades, respectively. **(B)** Metabolic fluxes in the central carbon metabolism in nmol/10^6^ cells/h (mean ± SD, *n* = 3). Blue arrows indicate anaplerotic and related reactions. Dotted lines assign enzymes and their net fluxes to the corresponding reactions. Total ATP and NADPH productions were calculated from all determined fluxes contributing to their direct or indirect generation or consumption. The metabolic efficiency was defined as the ratio of PDH and LDH fluxes. Abbreviations: ALT, alanine aminotransferase. ASNase, asparaginase; AST, aspartate aminotransferase; ATP, adenosine triphosphate; CS, citrate synthase; G6PDH, glucose-6-phosphate dehydrogenase; GA, glutamate anaplerosis, that is, α-ketoglutarate produced from Glu through glutamate dehydrogenase (GDH), ALT, and AST. GAPDH, glyceraldehyde-3-phosphate dehydrogenase. GS, glutamine synthase; IDH, isocitrate dehydrogenase; KDH, α-ketoglutarate dehydrogenase; LDH, lactate dehydrogenase; ME, malic enzyme; MDH, malate dehydrogenase; NADPH, nicotinamide adenine dinucleotide phosphate; PDH, pyruvate dehydrogenase; PPP, pentose phosphate pathway; PC, pyruvate carboxylase; PK, pyruvate kinase.

To provide first insights into the two aforementioned phenotypes, the uptake rates of AAs and BCKAs (Figure 2A; Supplementary Table 2) were explored as many AAs are not only involved in protein synthesis but also in central carbon metabolism and energy generation. Most AAs exhibited only moderately changed uptake following BCKA supplementation and, with few exceptions, presented slightly decreased influx for the detrimental 2KI/2KL condition. The most striking differences between the two phenotypes were observed with Glu and particularly Gln. The net influx of Glu decreased in the 2KI/2KL condition by 44% compared to the positive control, while Gln changed from a low uptake rate of +0.3 to a net excretion of −3.2 nmol/10^6^ cells/h. Aside from Glu and Gln, the increased uptake rates of L-asparagine (Asn, +41%) and L-aspartic acid (Asp, +35%) were other distinct features of the 2KI/2KL supplementation. Both Glu/Gln and Asp/Asn can be directly linked to the TCA cycle through anaplerosis, i.e., replenishing TCA cycle intermediates, such as α-ketoglutarate (KG) and oxaloacetate (OAA), ensuring sustained energy production. Furthermore, following 2KI/2KL supplementation, substantial export of glycine (Gly) was observed, in contrast to minimal Gly uptake in the other conditions. Although L-Alanine (Ala) did not differentiate the two phenotypes, any supplementation with BCKA changed the low excretion rate of Ala to net uptake, which may be linked to intracellular nitrogen availability.

The substitution of both or individual Ile and Leu with their KA derivatives resulted in reduced uptake of the replaced BCAA while simultaneously establishing the influx of the supplemented BCKAs. Consistent with previous studies (Schmidt et al., 2020; Reifenberg et al., 2025), valine’s keto acid (KV) was excreted at elevated rates in the 2KI/2KL condition, alongside a notable accumulation of Allo-Ile following supplementation of racemic KI. Overall, the AA uptake rates within the investigated time frame highlighted already distinct phenotypic responses to BCKA supplementation, not only concerning the BCAAs, but also other AAs, such as Gln, Glu, Asp, and Asn.

#### 3.1.3 BCKA supplementation reduces flux in ATP- and NADPH-generating pathways

The primary incentive of the MFA study was the determination of fluxes in glycolysis, PPP, and the TCA cycle in order to assess changes in total ATP and NADPH generation in response to BCKA supplementation. Multiple reactions in glycolysis and the TCA cycle generate ATP, both directly and indirectly through oxidative phosphorylation (Supplementary Table 3). The production of NADPH mainly arises from the activity of the PPP, represented by glucose-6-phosphate (G6PDH), and malic enzyme and malic enzyme (ME), which is part of the anaplerotic reactions (Supplementary Table 4). Both ATP and NADPH are essential for cell growth and survival, as they provide energy and reducing equivalents required for various metabolic processes.

The overall reaction rates in glycolysis and the TCA cycle reflected the two distinct phenotypes (Figure 2B). While equimolar replacement hardly changed the activities of glycolysis and the TCA cycle, the reaction rates in these pathways were substantially reduced in the 2KI/2KL condition. The glycolytic activity was 14% lower following supplementation with 2KI/2KL compared to the positive control. The reaction rates of hexokinase, glyceraldehyde-3-phosphate dehydrogenase, and pyruvate kinase represent this pathway in Figure 2B. Interestingly, L-Lactate dehydrogenase (LDH) activity appeared to be decoupled from glycolytic activity as the reduction of Pyr to lactate increased in response to BCKA supplementation. This finding, indicative of decreasing metabolic efficiency, was unexpected as lactate production is typically linked to the glycolytic flux in fast-proliferating cells (Mulukutla et al., 2016; Luginsland et al., 2024).

TCA cycle activity was consistent with glycolytic flux. Following 2KI/2KL supplementation, the flux through the TCA cycle decreased by approximately 20%, as indicated by reaction rates of citrate synthase and pyruvate, isocitrate, and α-ketoglutarate dehydrogenases. Since the majority of ATP is generated indirectly from NADH and reduced flavin adenine dinucleotide (FADH_2_) produced in the TCA cycle (Supplementary Table 3), the total ATP production was equally reduced by 20% in the case of the 2KI/2KL condition, which might explain the reduced cell growth and productivity.

Substantial differences between the two phenotypes were observed regarding anaplerosis (Figure 2B). The catabolism of Asn and Asp was elevated in response to 2KI/2KL supplementation, resulting in increased flux of carbons derived from these AAs into the TCA cycle (→ OAA). This finding aligns well with the increased uptake of Asn and Asp in this condition (Figure 2A). In contrast, glutamate anaplerosis (GA, Glu KG) was fully eliminated in this condition. GA is due to deamidation through glutamate dehydrogenase (GDH) and to aspartate and alanine aminotransferase (AST, ALT), which use Glu as amino group donor for transamination. The elevated rate of Asp transamination by AST resulted in increased KG consumption and Glu formation, thereby decreasing GA. While GDH activity remained unchanged (Supplementary Figure 1), decreased transamination of Pyr to Ala through ALT was observed following 2KI/2KL supplementation (Figure 2B), implying decreased formation of KG that may participate in the TCA cycle. Notably, while GA was eliminated, net Gln synthesis from Glu increased, corresponding with the intensified efflux of Gln. Up to this point, the anaplerotic contributions of AAs to the TCA cycle already provided valuable insights into the two phenotypes, including potential links between central carbon metabolism and the observed AA uptake rates. Nevertheless, the causal relationship leading to altered AST/ALT activity and elimination of GA could not be resolved by this experimental approach.

A more striking difference within the anaplerotic reactions involving Pyr was noted for ME and pyruvate carboxylase (PC). Following KI/KL and 2KI/2KL supplementation, the fluxes through these enzymes were reduced substantially. This reduction is particularly relevant because ME plays a crucial role in the overall production of NADPH. The total generation of NADPH, which is calculated by aggregating the reaction rates of ME and G6PDH (PPP), decreased gradually with increasing BCKA supplementation. Notably, despite a 29% decrease (2KI/2KL), this result should be interpreted with caution, considering the high standard deviation introduced by the G6PDH flux. Nevertheless, these findings suggest that the reduced cell growth and IgG production observed upon excessive BCKA supplementation might be linked to a reduced generation of ATP and NADPH within central carbon metabolism.

### 3.2 Simulated steady-state perfusion

The application of KI and KL with CHOK1 GS in fed-batch cultivation demonstrated that well-balanced KA supplementation can effectively slow down cell growth while enhancing *qP*. Although these effects compensated one another in the fed-batch process, preventing a substantially increased titer by the end of cultivation, this phenotype holds particular promise for steady-state perfusion. This mode of operation seeks to maintain a pre-defined cell density through continuous medium exchange while retaining the cells within the bioreactor. To counteract ongoing cell growth, once the steady-state condition is established, permanent removal of cell suspension from the bioreactor is required, causing a decreased harvest rate. Since the bleed is typically not processed further, steady-state perfusion may benefit from reduced cell growth by minimizing the bleed and improving product recovery in the harvest.

#### 3.2.1 Branched-chain keto acids decrease the bleed and increase specific productivity in simulated perfusion

(I) The potential use of KI and KL as bleed reducers and *qP* enhancers in perfusion and (II) the metabolic implications of their supplementation were investigated using two industrially relevant CHO cell lines. CHOK1 GS and CHO DG44 were evaluated in a simulated steady-state perfusion process with increasing KI or KL concentrations for 4 days. Given the cultivation in spin tubes, the process was considered “simulated” as the medium was exchanged one time daily by centrifugation. The target concentration of 20 × 10^6^ cells/mL was re-adjusted by manual bleeding of the cell suspension prior to the media exchange. Varying concentrations of KI or KL were spiked into commercially available Cellvento® 4CHO-X medium, which includes Ile and Leu.

To identify an appropriate range of KI and KL concentrations that inhibit cell growth without exhibiting toxicity, CHOK1 GS cells were exposed to a wide concentration range of individual BCKAs in a plate-based assay. Both KI and KL demonstrated growth inhibition starting in the low mM range with half-maximal effects at 5.5 and 4.5 mM for KI and KL, respectively (Supplementary Figure 2). Based on these findings, concentrations of 1 and 10 mM of either KI (LD_50_ 17.9 mM) or KL (LD_50_ 14.6 mM) were selected as lower and upper limits for further studies aimed at bleed reduction in perfusion without substantial cytotoxicity.

**FIGURE 3:**
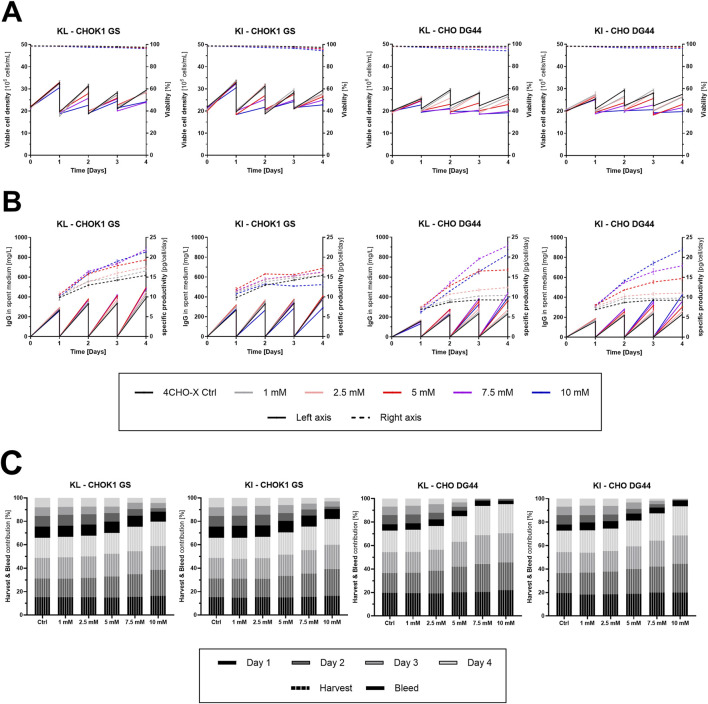
Viable cell density, viability, IgG titer, specific productivity, and harvest and bleed volumes obtained in simulated steady-state perfusion using CHOK1 GS and CHO DG44 cells supplemented with increasing concentrations of keto isoleucine and keto leucine. **(A)** Viable cell density in 10^6^ cells/mL (solid) and viability in percent (dashed) were determined using the Vi-CELL™ BLU cell counter (mean ± SD, *n* = 4). **(B)** IgG titer in mg/L (solid) was quantified using the Cedex Bio HT. Cell-specific IgG productivity in pg/cell/day (dashed) was calculated from the IgG in the spent medium on day *x* and the average viable cell density of day *x* and *x*–*1* (mean ± SD, *n* = 4). **(C)** Contribution of harvest (vertical lines) and bleed (solid fill) to the total volume exchanged during the 4-day process (in %) (mean, *n* = 4). The bleed was removed daily to maintain the target viable cell concentration of 20 × 10^6^ cells/mL; the remainder is regarded as the cell-free harvest.

For CHOK1 GS, results indicate substantially decreased cell growth and bleed volumes (Figures 3A,C) with increasing addition of KI or KL, in particular starting from the second cultivation day. The total bleed was reduced to a similar extent between KL and KI, increasing the harvest yield from 66% to 80% and 82%, respectively. *qP* enhancement was more striking for KL than KI (Figure 3B) with a maximal increase of 42% for 7.5 mM KL compared to the KA-free control. A concentration of 10 mM KL resulted in a slightly lower *qP* increase of 38%, while the same concentration of KI even led to a decrease in *qP* relative to the control. Considering the bleed as waste, the total amount of IgG recovered in the harvest during the 4 days of cultivation increased by 37% and 19% for KL and KI, respectively (Supplementary Figures 3A,B). Likewise, in the case of CHO DG44, both KAs led to substantially decreased cell growth (Figures 3A,C), enhancing the harvest yield from 73% to 95% with KL and 93% with KI. In contrast to CHOK1 GS, both KAs resulted in a strongly elevated *qP* (Figure 3B), achieving maximal enhancements of 147% and 139% for 7.5 mM KL and 10 mM KI, respectively, on day 4. The sum of IgG in the harvest increased by 98% with KL and 100% with KI (Supplementary Figures 3C,D).

Aside from reduced cell growth, the addition of KAs resulted in increased viable cell diameter (Supplementary Figure 4). This change in cell size is particularly important for perfusion culture, where the steady-state is maintained through online monitoring of biomass, which correlates with the viable cell volume (Bergin et al., 2022). To account for the increased cell volume following KA supplementation, the IgG productivity was corrected by the viable cell volume (Supplementary Figure 5). As a result, the *qP* gains were approximately halved. With CHOK1 GS, the productivity per cell volume was increased by 24% for 7.5 mM KL on day 4. In the case of CHO DG44, maximal enhancements were obtained with the addition of either 10 mM KL or KI, achieving 81% and 61%, respectively.

Ultimately, both KAs demonstrated clear benefits in simulated steady-state perfusion in terms of bleed reduction and *qP* enhancement, resulting in up to 37% (KL, CHOK1 GS) and 100% (KI, CHO DG44) more final biotherapeutic harvested during the process.

#### 3.2.2 Uptake and intracellular levels of alanine, glutamine, and glutamate are affected by KI and KL supplementation in steady-state perfusion

**FIGURE 4:**
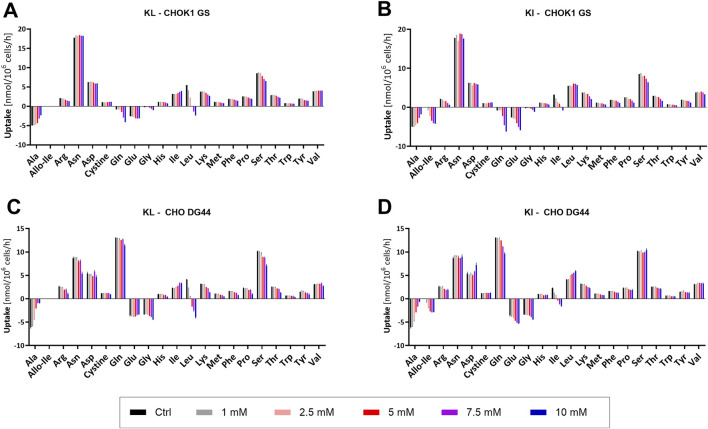
Amino acid uptake rates (in nmol/10^6^ cells/h) were determined during simulated steady-state perfusion of CHOK1 GS and CHO DG44 cells upon the addition of increasing concentrations of keto isoleucine (KI) and keto leucine (KL) (mean ± SD, n = 2). Addition of KL **(A)** or KI **(B)** during cultivation of CHOK1 GS and addition of KL **(C)** or KI **(D)** during cultivation of CHO DG44. Negative values indicate net excretion.

As demonstrated by the MFA, AA consumption and excretion can offer valuable insights into the observed phenotypes. AA quantification was performed with intra- and extracellular samples of the short-term perfusion experiment, and the net uptake rates were determined from the spent media samples as the average of all days (Figure 4). The intracellular content of each AA was normalized separately to the cell count, total protein content, or the viable cell volume. Independently of the normalization method, most AAs demonstrated higher intracellular levels upon BCKA supplementation, particularly in CHO DG44, indicating that normalization could not fully compensate for generally increased intracellular AA levels. To identify AAs whose behavior deviated from that trend, intracellular AA concentrations were determined as a percentage of the sum of all AAs (day 4, Supplementary Figure 6).

Since BCKAs were added to a medium containing BCAAs in the short-term perfusion experiments, the addition of the KAs increased the extracellular and intracellular pool of the corresponding AAs. The net uptake rate of the respective BCAA decreased, and net excretion was eventually observed at the highest BCKA concentrations. For instance, less Leu was consumed following KL supplementation, while the consumption of Ile was slightly increased. Interestingly, the uptake rate of Val remained mostly unchanged. Higher uptake was anticipated as a consequence of increased transamination between all three BCAAs and BCKAs (Reifenberg et al., 2025). Nevertheless, the intracellular content of Val decreased slightly relative to the other AAs. Furthermore, KV excretion and intracellular KV levels were substantially higher upon BCKA supplementation (Supplementary Figures 13E,F).

Ala excretion decreased substantially following BCKA supplementation, and the intracellular fraction decreased accordingly (Supplementary Figure 6), aligning well with the observations in the fed-batch process. Interestingly, only in the case of KL supplementation, a strong decrease in Pyr uptake was observed, even reaching net excretion (Supplementary Figure 7). In the case of CHO DG44, substantial pyruvate excretion was observed on the first day with KL > 5mM, which partially recovered to net uptake during cultivation, whereas the decreased pyruvate uptake in CHOK1 GS was persistent over time. In contrast to the phenotypes observed in fed-batch, the consumption rates of Asn and Asp did not change noticeably in the perfusion process. However, in the case of CHOK1 GS, KL supplementation led to decreased intracellular content of Asn, while both Asn and Asp levels decreased following KI supplementation. Gly (another AA, which clearly distinguished the two phenotypes in fed-batch) slightly increased excretion in perfusion, and noticeably elevated intracellular levels were observed upon KA supplementation with both cell lines.

In agreement with observations made in the fed-batch process, CHOK1 GS also excreted higher amounts of Gln following BCKA supplementation in perfusion. Moreover, substantially increased levels of this AA were detected intracellularly (Supplementary Figure 6). Interestingly, the addition of KI led to slightly higher excretion rates compared to KL at the same concentration, indicating different potencies of KI and KL regarding Gln production. Given the low GS activity in CHO DG44 cells, this cell line is unable to synthesize Gln and requires an exogenous supply. Aligning well with the increased export seen with CHOK1 GS, the consumption of Gln by CHO DG44 decreased slightly, particularly following KI addition, and intracellular concentrations were substantially elevated. In contrast to the net consumption of Glu by CHOK1 GS in the fed-batch process, both cell lines demonstrated net export of Glu under all conditions in the perfusion experiment (Figure 4). This discrepancy might be a consequence of the two different operation modes or media formulations used for the respective culture experiments. Nonetheless, the export of Glu was noticeably increased following KI supplementation aligning well with increased intracellular levels (Supplementary Figures 6B,D), indicating lower use for this AA upon KA supplementation.

Ultimately, the lower excretion of Ala and, more strikingly, the decreased intracellular use of Glu and Gln led to the conclusion that the effects on central carbon metabolism observed in the MFA study might similarly apply to the perfusion process. Additionally, the intra- and extracellular levels of Glu and Gln support the hypothesis that KI and KL influence metabolism with different potencies.

#### 3.2.3 Impact of KI and KL on the metabolome of CHOK1 GS and CHO DG44

To study the impact of KI or KL on the metabolome of the two CHO cell lines, spent media and cell pellets were collected daily during the short-term perfusion experiments and analyzed using an untargeted LC-MS approach. Peak selection and identification were performed using MetaboScape (Bruker Daltonics, Bremen, Germany) with the aid of an in-house spectral library. The number of features differed substantially between the different sample sets (Supplementary Table 5). A total of 380 compounds were identified as tiers 1–3. Features identified with tier 3 are marked with an asterisk (*) in the manuscript. All other features are classified as tier 1 or 1’, if not stated otherwise. Supplementary Table 6 is a list of all compounds mentioned in the manuscript containing abbreviations, IUPAC names, sum formulas, tiers, RTs, and *m/z* ratios. The AUC of each feature was calculated from the peak areas (normalized to the protein content). To explore the multidimensional data generated from these analyses, Pareto-scaled PLS regression including all features was performed for each individual combination of cell line (CHOK1 GS or CHO DG44), supplemented keto acid (KI or KL), MS source polarity (negative or positive), and sample type (extra- vs. intracellular). The AUC values of each feature and the KA concentrations were used as explanatory variable X and dependent variable Y, respectively. In addition to the loadings resulting from the PLS regressions, the ratios of minimal and maximal AUC values were used to discriminate features of higher interest. To facilitate the identification of compounds resulting from chemical interactions between KI/KL and other media components, the correlation coefficients between the peak areas of features detected in the media samples (extracellular, day 0) and the KI or KL concentrations were determined.

##### 3.2.3.1 Interactions of media components with KI and KL

Multiple features were identified in the samples of fresh media, which correlated well (*R* ^2^ > 0.8) with KI or KL concentrations, and the structures of the most abundant were elucidated. Features co-eluting (±0.1 min) with either KI or KL were not considered potential interactions as the change in abundance could not be distinguished from ionization effects during LC-MS analysis, i.e., adduct formation, ion suppression or enhancement. The following section highlights the most prominent interactions.

The formation of thiazolidines (Supplementary Figure 9A) through the condensation of cysteine and α-keto acids, such as Pyr (CP) and KG (CKG), is well known in cell culture media (Kuschelewski et al., 2017). In a similar manner, thiazolidines formed from both KL (CKL, Supplementary Figure 8A) and KI (CKI, Supplementary Figure 8B) were detected in the fresh media, correlating with the amount of supplemented KA. Following cultivation, the abundance of these compounds mostly decreased (except at high KI concentrations), suggesting consumption by the cells and maintained bioavailability of the cysteine moiety. Given the positive impact on media stability and process performance, which was previously demonstrated with CP and CKG, no detrimental effects were expected from the BCKA-containing thiazolidines.

Likewise, thiamine (vitamin B1) is known to interact with KAs, in particular by the formation of an adduct located at the C2 of the thiazolium ring, followed by decarboxylation of the KA moiety (Supplementary Figure 9B). This reaction occurs similarly to the enzymatic decarboxylation by the BCKA dehydrogenase (BCKDH) complex, involving thiamine pyrophosphate (ThPP) (Miller et al., 1962; Schnellbächer et al., 2019; Schnellbächer and Zimmer, 2023). The accumulation of hydroxyalkyl thiamines involving all three BCKAs (Th-KI, Th-KL, and Th-KV) was observed in extracellular samples (Supplementary Figure 10), yet not in the fresh media. Their identity was verified by incubating BCKAs with thiamine in aqueous solution at 37 °C [data not shown]. Although these compounds were not formed during storage of the media at 4 °C, the hydroxyalkyl thiamines were also regarded as interaction products as they are known to be formed through chemical reactions. Importantly, the addition of BCKAs did not affect the availability of thiamine itself (Supplementary Figure 11A), indicating that their absolute concentration is very low. Moreover, the formation of these compounds was considered non-critical to cell performance due to its similarity to the endogenous decarboxylation process.

Two compounds were identified that were exclusively found in KL-containing media and lacked the corresponding KI analogs in media containing KI. First, 2-isobutyl-4,5-dihydrothiazole-4-carboxylic acid (*, C_8_H_13_NO_2_S), which might be formed from degradation of CKL, was identified (Supplementary Figure 12A). This molecule was already present in the KL-supplemented media and increased following cultivation with either KI or KL. Second, a potential cross-aldol reaction product from of Pyr and KL was suggested (*, C_9_H_14_O_6_, Supplementary Figure 12B). This compound demonstrated substantially elevated abundances in the fresh media with KL, which diminished almost completely following cultivation. The impact of these two putative interaction products on the cell performance remains difficult to assess.

Finally, a significant interaction observed with media containing KI was the decreased levels of riboflavin (vitamin B2), an essential component of cell culture media (Supplementary Figure 11B). Supplementation of 10 mM KI reduced the relative riboflavin abundance by 64% compared to the control medium. Riboflavin is used in the metabolism of carbohydrates, AAs, and lipids for enzymatically controlled redox reactions (Schnellbächer et al., 2019). Given the semi-quantitative nature of untargeted metabolomics with LC-MS, the actual extent of this decrease needs to be interpreted with caution. Independent experiments verified that KI and KL substantially accelerated the degradation of riboflavin in aqueous solution [data not shown]. The most prominent degradation products resulting from riboflavin interaction with KI or KL were not detected in the current study, potentially due to the low abundance of riboflavin. The decreased availability of riboflavin might have an impact on cellular metabolism and overall culture performance.

Overall, the identified interaction products already provide insights into the compound classes, where significant changes in abundance need to be expected following cultivation with exogenous BCKAs.

##### 3.2.3.2 Metabolites directly derived from the BCAA/BCKAs

Multiple metabolites directly derived from the BCAAs or BCKAs demonstrated substantially altered abundance profiles following KI and KL supplementation. The abundances of the majority of these compounds correlated with the increased BCAA or BCKA pools (Supplementary Figure 13), independently of the two studied cell lines. For example, metabolites containing the Leu moiety, such as dipeptides, γ-glutamyl-Leu, or N-acetyl-Leu, demonstrated substantially elevated abundances upon KL supplementation (Supplementary Figure 14), hypothetically as a result of increased Leu availability following KL supplementation.

**FIGURE 5:**
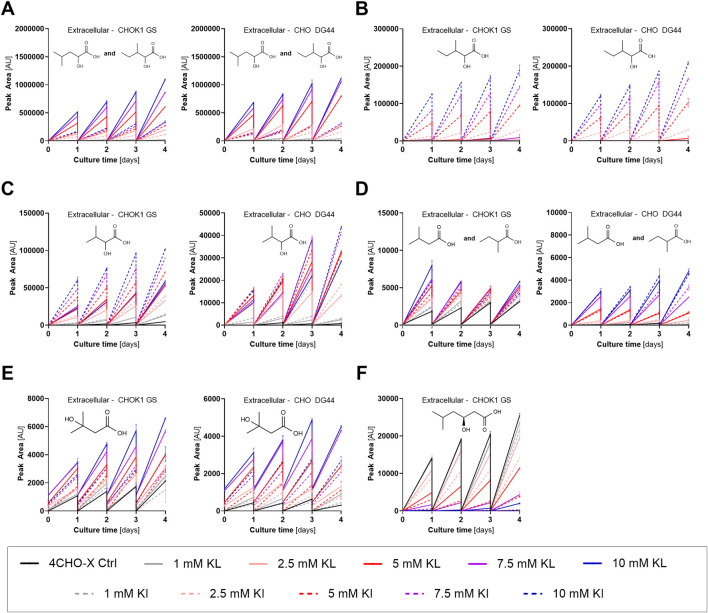
Abundance profiles of BCAA-derived metabolites, which were identified in spent media of simulated perfusion processes with CHOK1 GS and CHO DG44 (mean ± SD, *n* = 2). **(A)** α-Hydroxy acid of leucine (HL) and α-hydroxy acid of isoleucine (HI). Due to the close elution of the isomers, this feature is computed as a sum parameter of HI and HL. When the levels of HL strongly exceeded the HI levels, the peaks could not be integrated separately. **(B)** HI (when separation from HL peak was possible). **(C)** HV, 2-hydroxy-3-methylbutyric acid. **(D)** dcKI/dcKL, 3-methylbutanoic acid and 2-methylbutanoic acid, the decarboxylation products of keto leucine and keto isoleucine, respectively. Due to co-elution, this feature was determined as a sum parameter. **(E)** HMB, 3-hydroxyisovaleric acid, a by-product of Leu metabolism. **(F)** HMH, (3S)-3-hydroxy-5-methylhexanoic acid.

The formation of α-hydroxy acids (HA) from the BCKAs was already known from previous studies (Kuang et al., 2021; Ladiwala et al., 2023; Gonzalez et al., 2024; Reifenberg et al., 2025). Due to the presence of all three BCKAs, either through exogenous supply of KI and KL or through transamination leading to KV, elevated levels of all three BCHAs were anticipated in KA-supplemented conditions (Figures 5A–C). The accumulation of these HAs was considered non-critical to cell growth and productivity based on previous findings (Reifenberg et al., 2025).

The thiazolidines, CKL (Supplementary Figure 8A) and CKI (Supplementary Figure 8B), demonstrated correlation with the corresponding BCKAs, independently of the source of the KA, i.e., through exogenous supplementation or endogenous transamination. For example, following KL supplementation, CKL, which was already present in the medium, decreased upon cultivation, while CKI accumulated in parallel with the formation of KI. Intracellularly, a strong increase was observed for CKL and CKI following BCKA supplementation [data not shown]. Moreover, extra- and intracellular accumulation of two analogous metabolites of CKI and CKL, formed from the dipeptide Cys-Gly (potentially released from glutathione (GSH, γ-Glu-Cys-Gly) by γ-glutamyltransferase) rather than individual Cys, was observed (Gly-CKL, Gly-CKI, Supplementary Figures 8D,E). Eventually, the accumulation of KV led to the increased formation of the respective thiazolidine derivative (CKV, Supplementary Figure 8C), suggesting that the thiazolidine derivatives involving cysteine are in equilibrium with the available BCKAs.

In alignment with the observed formation of the hydroxyalkyl thiamines, (Th-KI, Th-KL, and Th-KV) in the spent media (Supplementary Figure 10), these compounds and the respective mono- (*) and diphosphate analogues (*) substantially increased intracellularly following BCKA supplementation [data not shown]. This accumulation concerned the derivatives of all three BCKAs and was anticipated based on the elevated KA levels and the observed, spontaneous reaction of thiamine with KAs at 37 °C. Notably, further similar metabolites were formed during the decarboxylation of Pyr (Th-Pyr) and KG (Th-KG) by the respective dehydrogenase complexes, which require ThPP as a co-factor, similar to the BCKDH complex. Although the intracellular abundance of Th-KG did not change notably [data not shown], the levels of Th-Pyr and its mono- and diphosphates (*, ThP-Pyr, ThPP-Pyr) decreased substantially with increasing KA supplementation with CHO DG44 (Supplementary Figure 15). A decrease was also observed in CHOK1 GS, but only ThPP-Pyr was detected. The decrease in ThPP-Pyr is particularly interesting, since Pyr decarboxylation is a crucial step in energy metabolism, indicating a potential bottleneck in the process connecting glycolysis and the TCA cycle.

Known by-products of KI and KL catabolism are isovaleric (dcKL) and 2-methylbutanoic acid (dcKI), which are formed by hydrolysis of their acyl-CoA derivatives following decarboxylation by the BCKDH complex (Gonzalez et al., 2024; Reifenberg et al., 2025). Despite the co-elution of dcKI and dcKL, substantially increased accumulation was observed extracellularly for this feature (Figure 5D) and its acyl-glucuronide [data not shown] as a result of BCKA supplementation. Likewise, another known by-product of Leu metabolism, 3-hydroxyisovaleric acid (HMB), increased substantially intra- and extracellularly, supporting the hypothesis of increased KL decarboxylation (Figure 5E). Further downstream of the Leu catabolism, the acyl-CoA of 3-hydroxy-3-methylglutaric acid was formed at the node linking Leu catabolism with the TCA cycle (through breakdown of the acyl-CoA into acetoacetate and acetyl-CoA) and terpenoid backbone biosynthesis (Kanehisa et al., 2025). The released 3-hydroxy-3-methylglutaric acid (HMG) was detected intracellularly and presented strong accumulation upon supplementation of both KI and KL (Supplementary Figures 16A,B), hypothetically caused by the hydrolysis of elevated 3-hydroxy-3-methylglutaryl-CoA. Unfortunately, acyl-CoA derivatives could not be analyzed with the applied LC-MS method due to unfavorable pH conditions for CoA derivatives during the LC separation.

One Leu-derived metabolite, namely, (3S)-3-hydroxy-5-methylhexanoic acid (HMH), which was only detected in the intra- and extracellular samples of CHOK1 GS, was particularly interesting due to its strong decrease upon KA supplementation (Figure 5F; Supplementary Figure 16C). 3-Hydroxy-5-methylhexanoic acid was detected in patients with isovaleric acidemia, where the deficiency of isovaleryl-CoA dehydrogenase activity leads to the accumulation of dcKL-CoA (Lehnert, 1981), yet neither the proposed formation of HMH from dcKL-CoA and acetyl-CoA nor the stereochemical composition was confirmed. Independent experiments in CHO cells with ^13^C-labeled Leu and glucose were carried out to elucidate the molecule’s (stereo-)isomeric composition. The analyses verified that (I) the metabolite contains two and five carbons from glucose and Leu, respectively; (II) C1 of Leu is the carbon that is not passed on to this metabolite; and (III) the feature solely consists of the Leu-, but not the Ile-derived analog. This information led to the conclusion that this metabolite was most likely formed by condensation of acetyl-CoA and dcKL-CoA (the product of Leu decarboxylation) or the released dcKL. Moreover, stereoisomer determination by LC–MS following derivatization using (+)-O,O′-diacetyl-L-tartaric anhydride (Reifenberg et al., 2025) demonstrated that only the 3S-enantiomer of this metabolite was formed [data not shown]. While the fatty acid synthase in the cytoplasm forms hydroxyalkyl intermediates in the 3R-conformation, the enzymes involved in the mitochondrial fatty acid oxidation and elongation machinery produce 3S-enantiomers. Therefore, the latter is more likely to be involved in the formation of (3S)-3-hydroxy-5-methylhexanoic acid. Overall, the decreased formation of this metabolite may indicate a higher demand or limited supply of acetyl-CoA as the availability of the dcKL-moiety was increased based on the elevated levels of dcKL/dcKI extra- and intracellularly.

##### 3.2.3.3 Non-BCAA metabolites impacted by KA supplementation

The supplementation of KI and KL also impacted the abundance of a multitude of metabolites, which are not directly derived from the BCAAs, but other AAs, such as α-hydroxy acids and N-acetyl- and γ-glutamyl-AAs (H-, NAc-, and γ-Glu-AA).

To begin with, the supplementation of BCKAs not only caused the accumulation of BCHAs but also affected the formation of α-hydroxy acids derived from other AAs, such as H-Trp, H-Phe, H-Met, H-Tyr, and H-Glu (Supplementary Figure 17). Individual H-AAs were previously associated with cell growth inhibition in CHO cells (Mulukutla et al., 2017). In the case of CHO DG44, the addition of KI strongly decreased the intra- and extracellular accumulation of H-Trp and H-Phe, while KL led to slightly increased formation of these compounds (Supplementary Figures 17B,D). The abundances of these metabolites did not change noticeably with CHOK1 GS for either KA (Supplementary Figures 17A,C). H-Met decreased strongly extra- and intracellularly upon KA supplementation with both cell lines (Supplementary Figures 17E,F). H-Tyr increased with both KAs in the case of CHOK1 GS, but only with KL in the case of CHO DG44 (Supplementary Figures 17G,H). α-Hydroxyglutaric acid (H-Glu) increased intracellularly with both KAs and cell lines, but the observed extracellular elevation was higher with KI compared to KL (Supplementary Figures 17I,J).

The abundances of multiple NAc-AAs increased intracellularly upon KA supplementation in both cell lines. Notably, more species were detected in CHO DG44 compared to CHOK1 GS (Supplementary Figure 18). The most striking increase was observed with NAc-Tyr with both cell lines and KAs, followed by NAc-Gln, yet the increase with CHO DG44 and KL was not as prominent (Supplementary Figures 18A,C). N6-Ac-Lys and NAc-Met were the only metabolites of this group, which demonstrated slightly lower levels following the addition of KI or KL in the case of CHOK1 GS (Supplementary Figures 18D,E). The intracellular content of NAc-Arg, -Ser, -Trp (only detected in CHO DG44), and -Ala did not change noticeably upon supplementation of KAs [data not shown].

A multitude of γ-glutamyl-AAs increased slightly upon KI or KL supplementation (Supplementary Figure 19), which might be formed following the transfer of the Glu moiety from glutathione (γ-Glu-Cys-Gly) to the accepting AA by membrane-bound γ-glutamyltransferase (Ikeda and Fujii, 2023). γ-Glu-Tyr, -Phe, and -Met accumulated substantially more in the spent media of CHOK1 GS resulting from the addition of KI and KL. The γ-Glu derivatives of Tyr, Trp*, Thr*, Phe, Met*, and Arg increased, at least slightly, extracellularly with both cell lines and KAs, and, in the case of γ-Glu-Ser, only with CHOK1 GS. Intracellularly, substantially elevated levels were observed for γ-Glu-Met, -Glu* and -Gln with CHO DG44, and slightly increased values were obtained for γ-Glu-Tyr, -Trp, -Phe, and -Lys* (α-N-acetylation) with both cell lines. The only detected γ-Glu-AA, which demonstrated decreased abundance following KA supplementation, was γ-Glu-Ala. Notably, the abundance of intracellular reduced glutathione (GSH) did not change markedly following BCKA supplementation. Oxidized glutathione (GSSG) only decreased moderately following KL supplementation in CHO DG44 [data not shown].

**FIGURE 6:**
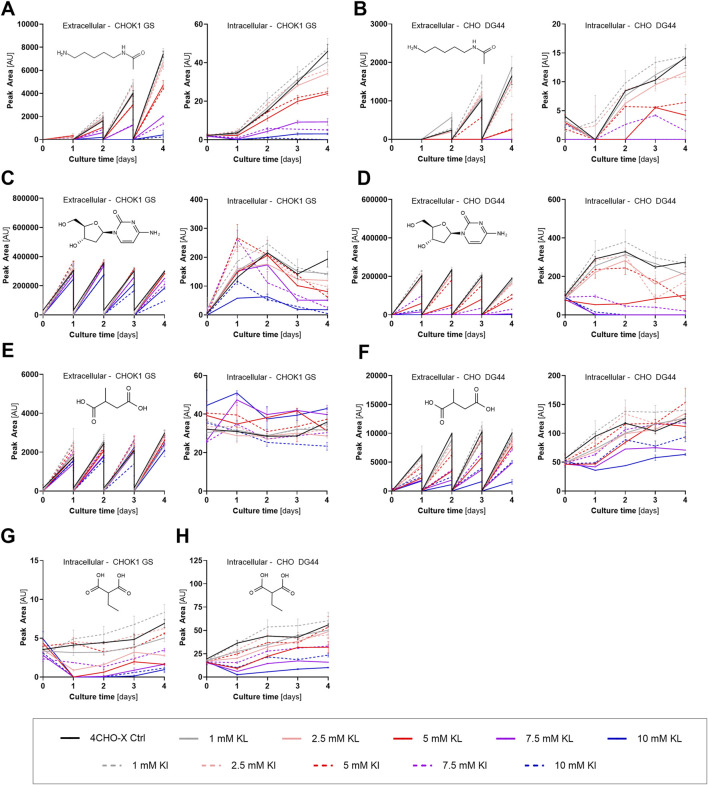
Abundance profiles of features, which decreased substantially upon the addition of keto isoleucine and keto leucine in simulated perfusion with either CHOK1 GS or CHO DG44 (mean ± SD, *n* = 2). Intracellular values were normalized to the total protein content of the extracted pellet. **(A)** N-Acetyl cadaverine (NAc-Cad) with CHOK1 GS. **(B)** NAc-Cad with CHO DG44. **(C)** 2’-Deoxycytidine (dCyt) with CHOK1 GS. **(D)** dCyt with CHO DG44. **(E)** Methyl succinic acid (MSA) with CHOK1 GS. **(F)** MSA with CHO DG44. **(G)** Ethylmalonic acid (EMA) with CHOK1 GS. **(H)** EMA with CHO DG44.

The most strikingly affected compounds, which were not classified as a group of AA-derived metabolites, are N-acetyl cadaverine (NAc-Cad), 2’-deoxycytidine (dCyt), methyl succinic acid (MSA), ethylmalonic acid (EMA), and CKG. NAc-Cad, a metabolite closely related to polyamines, which was suggested to enhance cell growth and production in CHO cells (Kang et al., 2023), was excreted by CHOK1 GS and CHO DG44 and, therefore, accumulated in the spent media of the control condition (Figures 6A,B). Following the addition of KI or KL, both the excretion and intracellular accumulation decreased and were almost fully eliminated with 10 mM KI or KL. Notably, another polyamine, N8-acetyl spermidine, did not change intracellularly and presented slightly increased values in the spent media samples [data not shown].

dCyt, a nucleoside and part of the DNA building block dCTP, accumulated extracellularly during cultivation in the control medium with both cell lines. This metabolite might be a product of dCTP breakdown through step-wise dephosphorylation. In the case of CHO DG44, the addition of KI or KL decreased and eventually fully eliminated this compound’s accumulation with 10 mM KA (Figure 6D). Moreover, intracellular levels of dCyt were also diminished and, at the highest KA concentrations, ultimately fully depleted. Intra- and extracellular abundances were also decreased with CHOK1 GS, yet not achieving full elimination (Figure 6C).

MSA has previously been associated with inhibition of cell growth (Kuang et al., 2021; Ladiwala et al., 2023). The supplementation of KAs decreased the accumulation of this metabolite, in particular with KL and CHO DG44 (Figure 6F). Intracellularly, lower abundances were also detected. Although the levels of MSA decreased slightly in the spent media of CHOK1 GS, a slight increase was observed intracellularly (Figure 6E). Hence, this metabolite presented different trends across the two cell lines. Nevertheless, the growth inhibition is unlikely to be induced by MSA due to the general decrease observed with this metabolite.

EMA was detected in the intracellular content of CHOK1 GS and CHO DG44. Following the addition of KI or KL, this metabolite demonstrated substantially decreased levels with both cell lines (Figures 6G,H). Different routes for the formation of EMA were suggested. In the most recent reports, EMA is thought to be produced from erroneous ATP-consuming carboxylation of butyryl-CoA by propionyl- or acetyl-CoA carboxylase (involved in lipid metabolism), followed by hydrolysis of the acyl-CoA (Corydon et al., 1996; Linster et al., 2011; Dewulf et al., 2021). A decrease in this metabolite according to this reaction might indicate reduced activity of the involved carboxylases or generally lower availability of ATP, butyryl-CoA or both. Alternatively, both EMA and MSA were suggested to be produced from abnormal (Allo-)Ile metabolism, in particular the dehydrogenation of (R)-2-methylbutyryl-CoA (decarboxylation product of (R)-KI) by short/branched chain specific acyl-CoA dehydrogenase, in ethylmalonic encephalopathy (Nowaczyk et al., 1998). Additionally, methylmalonyl-CoA mutase, which is also involved in Val and Ile metabolism, was reported to be capable of converting EMA-CoA into MSA-CoA (Taoka et al., 1994), indicating another potential link between the two metabolites and BCAA metabolism. Aside from decreased EMA formation, increased activity of ethylmalonyl-CoA decarboxylase, which functions to correct the erroneous carboxylation of butyryl-CoA, might contribute to the lower EMA levels (Linster et al., 2011; Dewulf et al., 2021). Acute administration of EMA was proposed to elicit oxidative stress in rat brain and skeletal muscle (Schuck et al., 2015). Levels of reactive oxygen species were unaffected by BCKA supplementation in CHO cells during fed-batch (Reifenberg et al., 2025), and considering the decreased EMA abundance in the current study, this metabolite is unlikely to contribute to oxidative stress and the consequent inhibition of cell growth. Nevertheless, the relationship between reduced EMA (and MSA) formation and decreased cell growth coupled with increased productivity remains unknown.

Similar to other KG-derived compounds, such as Glu, Gln, and α-hydroxyglutaric acid, the thiazolidine CKG increased strongly in the intracellular content of CHOK1 GS and CHO DG44 upon KA supplementation (Supplementary Figures 20A,B). In the cases of both cell lines, a higher increase was observed with KI than KL. This strong increase in CKG was unexpected, considering the hypothesized equilibrium of KAs and their thiazolidines and the fact that intracellular KG did not increase as strongly as the BCKAs following exogenous BCKA supplementation (Supplementary Figures 20C,D).

Ultimately, a variety of metabolites, which are not directly derived from the BCAAs and participate in different cellular processes, presented substantially changed abundances following exogenous supply of KI or KL. These variations suggest pathways that may need to be investigated to obtain further insights into the underlying mechanisms leading to decreased cell growth and enhanced productivity.

## 4 Discussion

In this work, the metabolism of CHO cells was investigated in response to the exogenous supply of KI and KL. First, the impact on central carbon metabolism was studied by ^13^C-MFA during the growth phase of a fed-batch with CHOK1 GS, replacing Ile and Leu by their KA sodium salts in the cell culture feed. Cell growth and IgG production rate suggested two distinct phenotypes for conditions with (I) equimolar replacement by KA and (II) supplementation in excess at two-fold concentrations. Equimolar replacement resulted in a moderate reduction in cell growth, which was compensated for by a slight increase in *qP*. In contrast, the condition supplemented with 2KI/2KL led to a strong decline in both growth rate and productivity. The uptake rates of multiple AAs and fluxes involving ATP and NADPH generation reflected this differentiation. Second, the use of BCKAs as a bleed reducer and *qP* enhancer was studied in simulated steady-state perfusion with CHOK1 GS and CHO DG44, supplementing KI or KL with increasing concentrations. Adding BCKAs effectively reduced the bleed required to maintain the target cell concentration and increased *qP*, additionally improving the IgG yield. An untargeted metabolomics approach via LC-MS was used to comprehensively explore the impact of BCKA supplementation on CHO metabolism, identifying multiple features positively or negatively correlated with the supplemented KA concentrations.

The use of KI and KL as bleed reducers and *qP* enhancers was evaluated using two industrially relevant CHO cell lines in simulated steady-state perfusion. The results suggest that BCKA supplementation may strongly increase the IgG yield in steady-state perfusion by (I) reducing the bleed and (II) increasing *qP*. However, the underlying mechanisms remain unclear. The observed reciprocal relationship between cell growth and productivity is commonly exploited in CHO processes by using a hypothermal shift or addition of small-chain fatty acids, such as butyric, valeric, or valproic acid. The increased productivity, combined with extended culture times, typically outweighs the reduced cell growth to increase the product yield in batch and fed-batch cultures (Liu et al., 2001; Wulhfard et al., 2010; Yang et al., 2014; Park et al., 2016; Coronel et al., 2016; Li et al., 2024; Galli et al., 2024). Cell cycle arrest in the G_0_/G_1_ phase is held responsible for the reduced cell growth and is particularly desirable due to the high metabolic activity and protein production in this phase (Pardee, 1989; Sunley and Butler, 2010; Park et al., 2016; Wolf et al., 2019). Notably, approaches to control cell cycle not only target cell cycle checkpoint regulators, i.e., cyclin-dependent kinases, but also impact other (signaling) pathways, and therefore lead to different cellular behavior (Du et al., 2015). Valproic and butyric acid were reported to be histone deacetylase inhibitors, hence impacting the cell cycle on an epigenetic level (Kramer, 2003; Sunley and Butler, 2010). Another common characteristic of the sustained arrest in the G_0_/G_1_ phase is the increased cell size (Kim et al., 2001; Bi et al., 2004; MacDonald et al., 2022), which was suggested to be positively correlated with *qP* in a previous study (Pan et al., 2017). Although cell cycle progression was not particularly investigated in the current study, the reduced cell growth, increased productivity, and larger diameters support the hypothesis that BCKA supplementation causes cell cycle arrest in CHO cells, similar to the aforementioned short-chain fatty acids. Ultimately, different approaches, such as proteomics and transcriptomics, might be required to gain further insights into the underlying mechanisms leading to the hypothesized cell cycle arrest observed with BCKA supplementation.

While KI and KL might be able to control cell growth themselves, the decarboxylation products of all three BCKAs, also classified as short-chain fatty acids, were found to decrease cell growth and increase *qP* in fed-batch cultures with CHO cells, potentially through inhibition of histone deacetylation (McBain et al., 1997; Mulukutla et al., 2017, 2019). Furthermore, catabolism of all three BCAAs was previously suggested to contribute to butyrate formation as BCAT1 knockout clones produced not only fewer decarboxylation products but also less butyrate than the respective wildtype (Harrington et al., 2021). These findings indicate that KI and KL might contribute to the cell cycle arrest through increased accumulation of butyrate resulting from elevated BCKA catabolism. Unfortunately, butyric acid was not detected within the current metabolomic study, preventing further evaluation of this hypothesis. However, in a previous study, dcKL and dcKI demonstrated decreased accumulation following BCKA supplementation in fed-batch and were consequently not thought to be linked to increased *qP* and decreased cell growth (Reifenberg et al., 2025). Further experiments are required to understand the opposing observations in terms of dcKI and dcKL accumulation and their relation to butyrate formation.

Limited studies exist on the use of cell cycle inhibitors in continuous processes with CHO cells (Du et al., 2015; Coronel et al., 2020; Avello et al., 2022), and only a few studies have specifically addressed the reduction of bleed by growth inhibition using additives in perfusion processes (Wolf et al., 2019). Valeric acid was found to inhibit CHO cell growth during the early phase of the steady-state, consequently reducing the bleed rate. However, the cells eventually recovered from the cell cycle arrest, returning to increased cell growth and bleed rate (Wolf et al., 2019). While this phenomenon may be less critical in batch and fed-batch processes, this outcome highlights the importance of ensuring sustained cell cycle control during extended perfusion cultivation. The short duration is a clear limitation of the current study as the cells might not be able to remain viable and sustain the high *qP*, especially at higher BCKA concentrations. Initial experiments with a cultivation time of 10 days using moderate KL concentrations in an advanced media formulation indicate that the BCKAs might effectively increase the IgG yield throughout a longer time period [data not shown]. Nevertheless, dedicated experiments are required to confirm the observed improvements in industrial processes and optimize the KA concentrations, balancing the beneficial *qP* enhancement and bleed reduction with sustaining a high viability.

^13^C-MFA was performed to understand the implications of BCKA supplementation on central carbon metabolism, specifically ATP and NADPH generation. Both compounds are essential for cell growth and survival, providing energy and reducing equivalents required for various metabolic processes. Total NADPH generation gradually decreased with increasing KA concentrations, and ATP production was particularly decreased in the 2KI/2KL condition. Therefore, the reduced cell growth might be linked to lower availability of reducing equivalents, i.e., NADPH. However, only the combination with decreased energy generation in the form of ATP might lead to the detrimental effects on cell growth and productivity observed with the 2KI/2KL condition. BCAA metabolism, including the BCKAs, was associated with altered central carbon metabolism across various health conditions and in insulin-responsive tissues (Yoon, 2016; Yao et al., 2023; Abdualkader et al., 2024; Bo and Fujii, 2024). Intracellular BCKAs were proposed to impair insulin signaling in cardiac and skeletal muscle by mTORC1 (mechanistic target of rapamycin complex 1) activation. mTOR is a central regulator of cell growth, proliferation, and metabolism in response to nutrients, growth factors, and cellular energy status (Szwed et al., 2021). Furthermore, mTOR is known to regulate glycolysis (Szwed et al., 2021), leading to decreased glucose uptake and mitochondrial oxygen consumption (Biswas et al., 2020). mTOR activation by BCAA-derived metabolites was also suggested in other studies to increase protein synthesis and inhibit protein breakdown (Wilkinson et al., 2013, 2018; Zhou et al., 2019; Reifenberg and Zimmer, 2024), which may be an explanation for the increase in *qP* observed in this study. In addition, the activation of mTOR has been reported to play an important role in sustained biomass growth of CHO cells following cell cycle arrest in the G_0_/G_1_ phase in a fed-batch process. This finding suggests a potential link between increased mTOR activation and the cell cycle arrest, which was hypothesized to occur following BCKA supplementation in the current study (Pan et al., 2019). Opposing the potential involvement of mTORC1 activation in the cell cycle arrest and biomass growth in this phase, mTORC1 activity was demonstrated to be lower in the G_1_ phase compared to the remaining cell cycle in HeLa cells (Joshi et al., 2024). Ultimately, dedicated investigations are necessary to elucidate the role of mTOR and insulin signaling in the cross-talk between BCAA metabolism, or particularly BCKAs, and glycolytic activity.

Since Pyr, the final product of glycolysis, fuels the TCA cycle, the decreased breakdown of glucose directly impacts the TCA cycle activity. Moreover, increased reduction of Pyr to lactate by LDH suggests a diversion of Pyr usage from energy-generating pathways, explaining that the flux through the TCA cycle was altered more (−20%) than glycolysis (−14%) in the 2KI/2KL condition compared to the control. A prerequisite for mitochondrial oxidation of Pyr is the transport from the cytosol through the mitochondrial pyruvate carrier, which was proposed to be inhibited by BCKAs in hepatocytes (Nishi et al., 2023). In early studies, BCKAs were reported to compromise PDH activity (Walajtys-Rode and Williamson, 1980; Jackson and Singer, 1983), but a recent report with isolated porcine PDH contradicts these findings, suggesting that the BCAAs themselves, rather than the BCKAs, inhibit PDH (Li et al., 2017). However, during the growth phase of CHO cells in fed-batch, the intracellular BCAA levels were decreased following the replacement of Ile and Leu with their KAs and, therefore, cannot be responsible for PDH inhibition (Reifenberg et al., 2025). Nevertheless, both reduced Pyr transport and PDH activity might limit the full oxidation of Pyr in the mitochondria. Decreased levels of intracellular ThPP-Pyr, an intermediate in the decarboxylation through PDH, which was detected in the metabolomics study, support the hypothesis that Pyr decarboxylation was also diminished in the perfusion process. Ultimately, compartment-specific MFA (Wijaya et al., 2021) may be required to resolve the impact of Pyr transport and altered PDH activity on the reduced TCA cycle flux.

The flux of malic enzyme, whose mitochondrial functions (ME2) are TCA cycle adjustment and generation of NADPH, was substantially reduced in KI/KL and 2KI/2KL conditions in fed-batch. Although equimolar replacement of both KA did not result in altered glycolytic flux, the reduced ME2 flux might be a foundation for effects observed with 2KI/2KL supplementation. AKT-mediated (protein kinase B) phosphorylation of ME2, which prevents its mitochondrial translocation and results in a previously unknown, cytoplasmic form of ME2, was associated with controlling glycolysis in HEK293T cells (Chen et al., 2024). This cytoplasmic ME2 facilitates the assembly of a high-molecular weight complex of the key enzymes phosphofructokinase, GAPDH, PK, and LDH, which demonstrated enhanced catalytic activity. This link between ME2 and glycolysis suggests that compromised ME2 activity, which was observed following BCKA supplementation in this study, may not be limited to impairing the mitochondrial functions of ME2 but may also have consequences for glycolytic capacity. These findings highlight the importance of investigating the impact of exogenous BCKA supplementation on ME2 expression, phosphorylation, and activity, along with its relationship to AKT signaling.

Interestingly, the reduced ME activity was observed specifically when KI was included in the feed. However, it remains unclear whether the impaired ME flux was caused specifically by KI or by generally increased KA supplementation, i.e., combining KI and KL. Although no reports were found directly linking BCAAs (and their metabolism) to ME activity, ME2 was proposed to regulate glutaminolysis. In detail, siRNA silencing of this enzyme strongly reduced the glutaminolytic flux in HCT116 cells (Jiang et al., 2013). In the perfusion process of the current study, supplementation of KI led to generally higher Gln excretion, indicating a hypothetical link between KI, reduced ME flux, and reduced/reversed anaplerosis through Glu/Gln. However, in fed-batch, only 2KI/2KL (not KI/KL) led to substantially increased Gln excretion, conflicting with the hypothesis that KI supplementation or reduced ME flux alone might cause the increased Gln production. Furthermore, the mechanism through which ME2 controls glutaminolysis remains unclear. Nevertheless, reducing ME2 expression ultimately led to substantial impairment of cell growth of U2OS and HCT116 cells (Jiang et al., 2013), highlighting the importance of ME activity for cell proliferation.

The uptake and excretion rates, along with intracellular levels of several AAs, such as Ala, Asn, Asp, Glu, and Gln, changed following BCKA supplementation. Notably, the observations differ slightly (I) between fed-batch and perfusion and (II) between the two CHO cell lines. As a result of BCKA supplementation, lower Gln utilization was observed in the form of increased synthesis and export (CHOK1 GS) or decreased uptake (CHO DG44). The fate of Gln might be tightly linked to lower TCA cycle flux and decreased Glu anaplerosis, i.e., lower contribution of Glu to KG and, therefore, higher Glu availability. In the case of CHOK1 GS, the uptake of Glu decreased in the 2KI/2KL condition in fed-batch and the excretion of this AA increased in perfusion, particularly in KI-supplemented conditions, where more Gln was exported. In addition to the bioenergetic role of Glu/Gln, an excess of intracellular Glu/Gln might be a result of decreased biosynthesis, for example, using the carbons or nitrogen to generate compounds, such as nucleotides, proteins, glutathione, hexosamines, and non-essential AAs (Curi et al., 2005; Cruzat et al., 2018; Yoo et al., 2020). Unfortunately, the metabolomic study did not conclusively pinpoint any of these pathways as being markedly impaired since only individual metabolites, such as the nucleoside 2’-deoxycytidine, presented substantial changes following BCKA supplementation.

In terms of bioenergetic needs, the anaplerotic contributions of Asp/Asn and Glu/Gln are known to be interconnected, enabling precise regulation of TCA cycle activity by replenishing the intermediates OAA and KG (Kirsch et al., 2022). Therefore, altered intracellular levels and fluxes of Asp, Asn, Glu, and Gln might be a result of re-adjusted anaplerotic needs. These AAs are linked through nitrogen transfers between Glu/KG and Asp/OAA. Similarly, Glu/KG is related to Ala/Pyr through transamination by ALT, a reaction whose flux was observed to slow down, generating less Ala and KG. Potentially, reduced Pyr availability, caused by decreased glycolysis and increased reduction to lactate, contributes to the increased Ala consumption. In human physiology, Gln, Glu, Ala, and BCAAs (and respective keto acids) are linked through an inter-organ cycle by transferring nitrogen from BCAAs or to BCKAs, depending on the organs’ requirements (Holeček, 2018; 2024). Although Glu and KG are common co-substrates of BCAT for BCAA/BCKA transamination, the current and previous studies suggest that BCAAs, rather than Glu, contribute to the amination of exogenously supplied BCKA in CHO cells (Schmidt et al., 2020; Reifenberg et al., 2025). Based on this assumption, it is unlikely that the balance of Glu and KG is directly impaired by transamination *via* BCAT following BCKA supplementation. Moreover, the increased Gln excretion supports the hypothesis that BCKA supplementation does not critically decrease intracellular nitrogen availability yet causes a shift in nitrogen disposal from Ala to Gln. It remains uncertain whether the altered uptake or excretion rates of these interconnected AAs are caused (I) directly by immediate interaction of BCKAs (or derived metabolites) with enzymes and metabolites in the anaplerotic pathways and transamination processes or (II) indirectly through the slowdown of glycolysis and, most importantly, the TCA cycle.

The metabolomics study indicated that BCKAs induce instability of riboflavin in fresh media, i.e., in the absence of cells and despite storage at 4 °C. In independent experiments, CHO cells did not survive pre-passaging in riboflavin-depleted medium [data not shown], indicating the general necessity of this vitamin. In contrast, fed-batch experiments with riboflavin-containing medium and riboflavin-depleted feeds demonstrated normal cell growth, although the vitamin was depleted mid-growth phase [data not shown], suggesting that the cells might maintain normal growth behavior despite low riboflavin availability. Although riboflavin was not completely degraded in the perfusion media and cell growth might not be disturbed by low riboflavin levels, the BCKA-induced degradation requires further investigations.

Following KI and KL supplementation, several interaction products and metabolites, such as HMG and dcKI/dcKL, increased strongly and disturbances in metabolic processes connected to downstream metabolites cannot be excluded. For example, HMG-CoA is reduced to mevalonic acid, a precursor of cholesterol, with the consumption of two NADPH molecules (Burg and Espenshade, 2011). Hence, increased HMG(-CoA) levels might indicate (I) lower activity of the mevalonate pathway, limiting the synthesis of cholesterol, or (II) impaired catabolic breakdown of HMG-CoA into acetyl-CoA and acetoacetate but might also be solely caused by (III) increased KL catabolism, leading to the accumulation of HMG(-CoA). Among the metabolites formed from BCKAs, HMH stood out because this metabolite was markedly decreased despite the increased dcKI/dcKL abundance. Potentially, HMH formation is limited by its second building block acetyl-CoA, which supports the hypothesis of decreased PDH activity, e.g., through decreased glycolysis and metabolic efficiency. Notably, the metabolite was not detected in CHO DG44; hence, it is not indicative of the different phenotypes in this cell line. Eventually, MFA with ^13^C-labeling of individual BCKAs or BCAAs might provide deeper insights into the dynamics of pathways, which are directly connected to BCKA catabolism, as further interpretation of the altered metabolite levels is limited by the static nature of metabolomics approaches.

Multiple metabolites, which were not derived from BCKAs, presented substantially altered abundance profiles following BCKA supplementation. For example, the formations of individual AA derivatives, such as H-AA, NAc-AA, and γ-Glu-AAs, were affected. Furthermore, metabolites that are not as closely related to AAs, such as NAc-Cad, dCyt, MSA, and EMA, decreased strongly, resulting from exposure of CHO cells to exogenous BCKAs. MSA and EMA were associated with abnormal Ile metabolism in individuals suffering from ethylmalonic encephalopathy (Nowaczyk et al., 1998), and MSA was reported to inhibit the growth of CHO cells (Kuang et al., 2021; Ladiwala et al., 2023). However, not enough is known about the role of these metabolites and the consequences of their reduced formation, limiting the conclusions that can be drawn with respect to the observed phenotypes in perfusion. Therefore, further insights into the pathways involving these metabolites are necessary to understand their relation to BCKA supplementation, i.e., whether the changes in metabolism are a cause or a consequence of the intracellular events leading to altered cell growth and specific productivity. These investigations may include proteomics, transcriptomics, or MFA studies in extended steady-state perfusion processes with BCKA supplementation for bleed reduction and *qP* enhancement.

## 5 Conclusion

This study demonstrates that carefully dosed KI and KL can be beneficial across different operation modes in biotechnological processes, although at first sight the improvements in product yield appear substantially higher in steady-state perfusion than in fed-batch. Aside from controlling growth rate and *qP*, KAs enable higher concentration of media and feeds through their enhanced solubility, not only opening new possibilities for process intensification but also simplifying media preparation and storage. Additionally, the findings underscore that MFA and metabolomics studies yield valuable yet distinct insights into the metabolic impact and fate of novel media components.

## Data Availability

The original contributions presented in the study are included in the article/Supplementary Material; further inquiries can be directed to the corresponding author.
