# Supplementary material for: Impact of keto leucine and isoleucine on CHO cell central carbon metabolism and performance in fed-batch and steady-state perfusion
Source: Front Bioeng Biotechnol. 2026 Feb 17;14:1708088. doi: 10.3389/fbioe.2026.1708088 (PMC12972755; doi:10.3389/fbioe.2026.1708088)
Supplement: Supplementary file 1 [file DataSheet1.pdf]

Supplementary Material

1 Fed-batch / Metabolic Flux Analysis

Supp. Table 1: Individual reactions of metabolic network

Pathway	Name	Reaction
**AA Metabolism**	GLNsynthetase	GLU + NH3 ↔ GLN
**AA Metabolism**	SERpyruvateT	3PG + NH3 ↔ SER_c
**AA Metabolism**	ALA_aminotransferase	ALA ↔ Pyruvate_c
**AA Metabolism**	THRmet	THR → CO2 + Pyruvate_m + NH3
**AA Metabolism**	ASPaminotransferase	OAA ↔ ASP
**AA Metabolism**	ASNtransferase	ASP + NH3 ↔ ASN
**AA Metabolism**	PROoxidase	PRO ↔ GLU
**AA Metabolism**	CYS TAA	CYS → Pyruvate_c + NH3
**AA Metabolism**	4Monooxygenase	PHE → TYR
**AA Metabolism**	TRPmet	TRP → aKetoadipate + CO2 + CO2 + ALA + NH3
**AA Metabolism**	Akgmet	aKetoadipate → ACCOA_m + ACCOA_m + CO2 + CO2
**AA Metabolism**	TYRmet	TYR → Fumarate + ACCOA_m + ACCOA_m + CO2 + NH3
**AA Metabolism**	LYSmet	LYS → aKetoadipate
**AA Metabolism**	ARGinase	ARG → GLU + Urea + NH3
**AA Metabolism**	KL_met	KL → ACCOA_m + ACCOA_m + ACCOA_m
**AA Metabolism**	KV_met	KV → Propionyl_CoA + CO2 + CO2
**AA Metabolism**	KI_met	KI → Propionyl_CoA + ACCOA_m + CO2
**AA Metabolism**	ILE_KL_TA	ILE + KL ↔ LEU + KI
**AA Metabolism**	ILE_KV_TA	ILE + KV ↔ VAL + KI
**AA Metabolism**	LEU_KV_TA	LEU + KV ↔ VAL + KL
**AA Metabolism**	KL_red	KL → HL
**AA Metabolism**	KV_red	KV → HV
**AA Metabolism**	KI_red	KI → HI
**AA Metabolism**	CYST_DEG	CYST ↔ CYS + CYS
**AA Metabolism**	LEU_Met	LEU → KL
**AA Metabolism**	ILE_Met	ILE → KI
**AA Metabolism**	VAL_Met	VAL → KV
**AA Metabolism**	LEU_KI_TA	LEU + KI → Allo_Ile + KL
**AA Metabolism**	KI_VAL_TA	KI + VAL → Allo_Ile + KV
**Anaplerosis**	MalicEnzyme	Malate → CO2 + Pyruvate_c
**Anaplerosis**	PyruvateC	Pyruvate_m + CO2 → OAA
**Anaplerosis**	PropionylCoACarboxylase	Propionyl_CoA + CO2 → Succinate
**Anaplerosis**	Glutamate/Ketoglutarate Anaplerosis	GLU ↔ AKG_m + NH3
**Glycolysis**	Hexokinase	Glucose → G6P
**Glycolysis**	PyruvateK	PEP → Pyruvate_c
**Glycolysis**	Phosphofructokinase	FructoseBP → DHAP + G3P
**Glycolysis**	GAPDH	G3P ↔ 3PG
**Glycolysis**	Triose phosphate isomerase	DHAP ↔ G3P
**Glycolysis**	PhosphoglucoseI	G6P ↔ Fructose6P
**Glycolysis**	FructoseBP	FructoseBP ↔ Fructose6P
**Glycolysis**	pgm	3PG ↔ PEP
**Glycolysis**	LactateDH	Pyruvate_c ↔ Lactate
**Glycolysis**	Pyruvate_Ex	Pyruvate_c ↔ Pyruvate_m
**OPPP**	Glucose6PDH	G6P → CO2 + Pentose5P
**TCA cycle**	PyruvateDH	Pyruvate_m → ACCOA_m + CO2

Pathway	Name	Reaction
**TCA cycle**	CitrateS	ACCOA_m + OAA → Citrate
**TCA cycle**	IsocitrateDH	Citrate ↔ AKG_m + CO2
**TCA cycle**	AkgDH	AKG_m → Succinate + CO2
**TCA cycle**	SuccinateDH	Succinate ↔ Fumarate
**TCA cycle**	Fumarase	Fumarate ↔ Malate
**TCA cycle**	MalateDH	Malate ↔ OAA
**TCA cycle**	CitrateL	Citrate → ACCOA_c + OAA
**Transport**	CO2_EX	CO2 → CO2_EX
**Transport**	CO2source	CO2_source → CO2
**Transport**	LYS_IN	LYS.Media → LYS
**Transport**	THR_IN	THR.Media → THR
**Transport**	TRP_IN	TRP.Media → TRP
**Transport**	PHE_IN	PHE.Media → PHE
**Transport**	TYR_IN	TYR.Media ↔ TYR
**Transport**	VAL_IN	VAL.Media → VAL
**Transport**	LEU_IN	LEU.Media → LEU
**Transport**	ILE_IN	ILE.Media → ILE
**Transport**	HIS_IN	HIS.Media → HIS
**Transport**	MET_IN	MET.Media → MET
**Transport**	SER_IN	SER.Media ↔ SER_c
**Transport**	GLY_IN	GLY.Media ↔ GLY
**Transport**	ARG_IN	ARG.Media ↔ ARG
**Transport**	ASP_IN	ASP.Media ↔ ASP
**Transport**	CYS_IN	CYS.Media ↔ CYS
**Transport**	GLU_IN	GLU.Media ↔ GLU
**Transport**	GLN_IN	GLN.Media ↔ GLN
**Transport**	PRO_IN	PRO.Media ↔ PRO
**Transport**	ASN_IN	ASN.Media ↔ ASN
**Transport**	Glucose_L	Glucose.Labeled → Glucose.Media
**Transport**	Glucose_U	Glucose.Unlabeled → Glucose.Media
**Transport**	Lactate_IN	Lactate.Media ↔ Lactate
**Transport**	ALA_IN	ALA.Media ↔ ALA
**Transport**	Glucose_IN	Glucose.Media → Glucose
**Transport**	AMM_IN	NH3.Media ↔ NH3
**Transport**	KI_IN	KI.Media ↔ KI
**Transport**	KV_IN	KV.Media ↔ KV
**Transport**	KL_IN	KL.Media ↔ KL
**Transport**	HL_IN	HL.Media ↔ HL
**Transport**	HV_IN	HV.Media ↔ HV
**Transport**	HI_IN	HI.Media ↔ HI
**Transport**	CYST_IN	CYST.Media → CYST
**Transport**	PYR_IN	Pyruvate_c.Media ↔ Pyruvate_c
**Transport**	Allo-Ile_IN	Allo_Ile.Media ↔ Allo_Ile
**Biosynthetic**	Biomass	111*G6P + 49*DHAP + 77.2*GLU + 77.2*GLN + 73.0*THR + 68.9*Pentose5P + 442.8*ACCOA_c + 59.9*ASP + 59.9*ASN + 68.0*PRO + 68.8*CYS + 116.6*GLY + 27.5*HIS + 48.1*PHE + 39.1*TYR + 20.7*TRP + 62.4*ILE + 113.7*LEU + 86.5*VAL + 100.5*LYS + 40.9*MET + 69.6*ARG + 113.1*ALA + 85.8*SER → **Biomass**
**Biosynthetic**	mAb	70*GLU + 68*GLN + 110*THR + 56*ASP + 56*ASN + 92*PRO + 34*CYS + 90*GLY + 22*HIS + 44*PHE + 64*TYR + 26*TRP + 30*ILE + 120*LEU + 130*VAL + 92*LYS + 16*MET + 40*ARG + 74*ALA + 176*SER → **mAb**

Supp. Table 2: Net fluxes of Metabolic Flux Analysis showing the values with their respective standard deviation (SD).

Pathway	Reaction	Ile/Leu		Ile/KL		KI/KL		2KI/2KL	
		Net fluxes [nmol/10^6 cells/h]							
		**Value**	SD	**Value**	SD	**Value**	SD	**Value**	SD
**AA Metabolism**	GLNsynthetase	**1.15**	0.03	**0.93**	0.05	**0.96**	0.05	**4.13**	0.12
**AA Metabolism**	SERpyruvateT	**-5.80**	0.09	**-6.09**	0.08	**-5.87**	0.21	**-5.09**	0.11
**AA Metabolism**	ALA_aminotransferase	**-2.08**	0.09	**-1.19**	0.08	**-0.57**	0.09	**-0.17**	0.06
**AA Metabolism**	THRmet	**0.95**	0.04	**1.00**	0.05	**0.94**	0.11	**0.71**	0.08
**AA Metabolism**	ASPaminotransferase	**-5.26**	0.11	**-5.60**	0.10	**-5.63**	0.19	**-8.98**	0.04
**AA Metabolism**	ASNtransferase	**-2.40**	0.10	**-2.46**	0.04	**-2.47**	0.11	**-4.27**	0.02
**AA Metabolism**	PROoxidase	**0.89**	0.04	**0.83**	0.05	**0.83**	0.10	**0.67**	0.08
**AA Metabolism**	CYS_TAA	**0.33**	0.04	**0.49**	0.06	**0.52**	0.07	**1.29**	0.03
**AA Metabolism**	SERhydroxyT	**-0.71**	0.03	**-0.66**	0.05	**-0.71**	0.06	**-0.84**	0.02
**AA Metabolism**	Histidase	**0.32**	0.01	**0.33**	0.02	**0.29**	0.04	**0.26**	0.03
**AA Metabolism**	GLYsynthase	**1.16**	0.01	**1.15**	0.02	**1.15**	0.03	**1.30**	0.02
**AA Metabolism**	4Monooxygenase	**0.76**	0.03	**0.80**	0.03	**0.71**	0.07	**0.68**	0.04
**AA Metabolism**	TRPmet	**0.41**	0.01	**0.46**	0.01	**0.40**	0.03	**0.45**	0.03
**AA Metabolism**	Akgmet	**1.49**	0.05	**1.57**	0.08	**1.38**	0.13	**1.27**	0.08
**AA Metabolism**	TYRmet	**0.51**	0.05	**0.53**	0.08	**0.37**	0.12	**0.15**	0.04
**AA Metabolism**	LYSmet	**1.07**	0.05	**1.11**	0.07	**0.98**	0.12	**0.83**	0.07
**AA Metabolism**	METmet	**0.13**	0.02	**0.16**	0.02	**0.15**	0.04	**0.19**	0.02
**AA Metabolism**	ARGinase	**0.65**	0.04	**0.66**	0.04	**0.58**	0.08	**0.46**	0.05
**AA Metabolism**	KL_met	**2.13**	0.06	**2.25**	0.11	**1.98**	0.15	**1.42**	0.34
**AA Metabolism**	KV_met	**1.61**	0.05	**1.71**	0.06	**1.45**	0.15	**0.74**	0.15
**AA Metabolism**	KI_met	**$1.5x10^{-7}$**	0.48	**$1.8x10^{-7}$**	0.37	**1.72**	0.11	**1.36**	0.45
**AA Metabolism**	ILE_KL_TA	**-0.29**	NA	**0.45**	NA	**0.73**	NA	**-0.13**	NA
**AA Metabolism**	ILE_KV_TA	**-0.64**	NA	**0.45**	NA	**-0.85**	NA	**0.04**	NA
**AA Metabolism**	LEU_KV_TA	**1.83**	NA	**-1.46**	NA	**-0.47**	NA	**-1.61**	NA
**AA Metabolism**	KL_red	**0.08**	0.004	**0.30**	0.01	**0.60**	0.01	**1.09**	0.003
**AA Metabolism**	KV_red	**0.03**	0.001	**0.17**	0.002	**0.38**	0.01	**0.40**	0.004
**AA Metabolism**	KI_red	**0.01**	0.001	**0.04**	0.002	**0.08**	0.002	**0.13**	0.002
**AA Metabolism**	CYST_DEG	**0.29**	0.01	**0.31**	0.001	**0.30**	0.01	**0.31**	0.01
**AA Metabolism**	LEU_Met	**0.11**	0.54	**$1.8x10^{-7}$**	0.43	**0.57**	0.27	**0.10**	0.09
**AA Metabolism**	ILE_Met	**2.91**	1.25	**0.88**	0.60	**0.56**	0.27	**0.07**	0.08
**AA Metabolism**	VAL_Met	**1.18**	0.83	**0.60**	0.53	**0.56**	0.27	**0.45**	0.18
**AA Metabolism**	LEU_KI_TA	**0.08**	0.04	**1.69**	0.46	**0.55**	0.15	**$1.x10^{-7}$**	0.10
**AA Metabolism**	KI_VAL_TA	**1.75**	0.46	**0.20**	0.08	**0.34**	0.10	**1.29**	0.34
**Anaplerosis**	MalicEnzyme	**29.89**	5.67	**36.76**	3.34	**14.36**	0.91	**11.11**	0.97
**Anaplerosis**	PyruvateC	**17.09**	5.49	**22.95**	3.36	**$1.9x10^{-7}$**	0.63	**$1.4x10^{-7}$**	0.64

Pathway	Reaction	Ile/Leu		Ile/KL		KI/KL		2KI/2KL	
		Net fluxes [nmol/10^6 cells/h]							
		**Value**	SD	**Value**	SD	**Value**	SD	**Value**	SD
**Anaplerosis**	PropionylCoACarboxylase	**1.74**	0.52	**1.87**	0.42	**3.32**	0.21	**2.29**	0.47
**Anaplerosis**	Glutamate/Ketoglutarate Anaplerosis	**5.29**	0.19	**5.81**	0.25	**5.05**	0.35	**-0.31**	0.25
**Biosynthesis**	Biomass	**0.016**	0.00029	**0.015**	0.00057	**0.014**	0.00051	**0.0092**	0.000055
**Biosynthesis**	mAb	**0.0035**	0.000059	**0.0038**	0.000060	**0.0035**	0.000062	**0.0027**	0.000017
**Glycolysis**	Hexokinase	**45.53**	0.49	**45.59**	0.94	**43.72**	1.03	**39.04**	0.25
**Glycolysis**	PyruvateK	**88.68**	1.69	**91.25**	2.21	**85.22**	2.06	**76.51**	1.33
**Glycolysis**	Phosphofructokinase	**41.00**	1.22	**42.94**	1.35	**38.86**	1.43	**34.47**	1.28
**Glycolysis**	GAPDH	**82.89**	1.56	**85.16**	2.20	**79.35**	2.01	**71.42**	1.33
**Glycolysis**	Triose_phosphate_isomerase	**40.22**	1.32	**42.22**	1.36	**38.16**	1.44	**34.02**	1.28
**Glycolysis**	PhosphoglucoseI	**37.68**	3.39	**42.94**	2.69	**34.20**	3.54	**28.63**	3.82
**Glycolysis**	FructoseBP2	**-41.00**	1.22	**-42.94**	1.35	**-38.86**	1.43	**-34.47**	1.28
**Glycolysis**	pgm	**88.68**	1.69	**91.25**	2.21	**85.22**	2.06	**76.51**	1.33
**Glycolysis**	LactateDH	**1.60**	0.37	**2.85**	0.07	**4.42**	0.33	**11.53**	0.43
**Glycolysis**	Pyruvate_Ex	**116.55**	6.47	**125.78**	4.37	**96.62**	2.52	**79.97**	1.52
**OPPP**	Glucose6PDH	**6.08**	3.52	**1.01**	2.20	**7.95**	3.79	**9.39**	3.82
**TCA cycle**	PyruvateDH	**100.40**	2.21	**103.83**	2.31	**97.55**	2.39	**80.68**	1.33
**TCA cycle**	CitrateS	**110.78**	2.63	**114.78**	2.57	**108.71**	2.72	**89.15**	1.84
**TCA cycle**	IsocitrateDH	**103.73**	2.83	**108.77**	2.68	**102.07**	2.86	**85.91**	1.85
**TCA cycle**	AkgDH	**109.02**	2.74	**114.58**	2.81	**107.12**	3.04	**85.59**	2.07
**TCA cycle**	SuccinateDH	**110.76**	3.22	**116.45**	3.03	**110.43**	3.13	**87.89**	2.11
**TCA cycle**	Fumarase	**111.27**	3.26	**116.98**	3.06	**110.80**	3.18	**88.03**	2.32
**TCA cycle**	MalateDH	**81.38**	5.32	**80.22**	3.85	**96.44**	2.81	**76.92**	1.94
**TCA cycle**	CitrateL	**7.05**	0.13	**6.01**	0.23	**6.64**	0.24	**3.24**	0.02
**Transport**	ALA_IN	**-0.44**	0.08	**0.30**	0.02	**0.89**	0.05	**0.56**	0.05
**Transport**	Allo_Ile_IN	**NA**	NA	**NA**	NA	**-0.90**	0.02	**-1.29**	0.02
**Transport**	AMM_IN	**-16.76**	0.33	**-17.78**	0.52	**-16.45**	0.60	**-15.39**	0.29
**Transport**	ARG_IN	**1.90**	0.03	**1.87**	0.02	**1.76**	0.07	**1.22**	0.05
**Transport**	ASN_IN	**3.55**	0.10	**3.58**	0.03	**3.57**	0.11	**4.98**	0.02
**Transport**	ASP_IN	**4.02**	0.03	**4.26**	0.07	**4.25**	0.14	**5.42**	0.04
**Transport**	CYS_IN	**0.83**	0.01	**0.86**	0.01	**0.86**	0.02	**1.21**	0.003
**Transport**	CYST_IN	**0.29**	0.01	**0.31**	0.001	**0.30**	0.01	**0.31**	0.01
**Transport**	GLN_IN	**0.32**	0.01	**0.48**	0.01	**0.45**	0.02	**-3.16**	0.12
**Transport**	GLU_IN	**6.06**	0.15	**6.35**	0.15	**5.73**	0.28	**3.40**	0.20
**Transport**	Glucose_IN	**45.53**	0.49	**45.59**	0.94	**43.72**	1.03	**39.04**	0.25
**Transport**	GLY_IN	**0.30**	0.004	**0.30**	0.01	**0.24**	0.01	**-0.71**	0.03
**Transport**	HI_IN	**-0.01**	0.001	**-0.04**	0.002	**-0.08**	0.002	**-0.13**	0.002
**Transport**	HIS_IN	**0.83**	0.01	**0.83**	0.002	**0.78**	0.04	**0.58**	0.03
**Transport**	HL_IN	**-0.08**	0.004	**-0.30**	0.01	**-0.60**	0.01	**-1.09**	0.003

Pathway	Reaction	Ile/Leu		Ile/KL		KI/KL		2KI/2KL	
		Net fluxes [nmol/10^6 cells/h]							
		**Value**	SD	**Value**	SD	**Value**	SD	**Value**	SD
**Transport**	HV_IN	**-0.03**	0.001	**-0.17**	0.002	**-0.38**	0.01	**-0.40**	0.004
**Transport**	ILE_IN	**3.08**	0.03	**2.86**	0.02	**1.45**	0.09	**0.62**	0.07
**Transport**	KI_IN	**-0.14**	0.01	**0.15**	0.05	**2.26**	0.06	**2.81**	0.45
**Transport**	KL_IN	**-0.10**	0.01	**2.76**	0.09	**2.66**	0.07	**3.89**	0.34
**Transport**	KV_IN	**-0.10**	0.003	**0.07**	0.02	**-0.40**	0.005	**-2.17**	0.13
**Transport**	Lactate_IN	**-1.60**	0.37	**-2.85**	0.07	**-4.42**	0.33	**-11.53**	0.43
**Transport**	LEU_IN	**4.53**	0.04	**1.95**	0.01	**2.01**	0.12	**$1.4x10^{-7}$**	0.04
**Transport**	LYS_IN	**3.00**	0.04	**2.97**	0.03	**2.78**	0.11	**2.00**	0.07
**Transport**	MET_IN	**0.83**	0.01	**0.82**	0.01	**0.78**	0.03	**0.61**	0.02
**Transport**	PHE_IN	**1.68**	0.02	**1.70**	0.01	**1.59**	0.06	**1.26**	0.04
**Transport**	PRO_IN	**2.29**	0.03	**2.33**	0.01	**2.21**	0.10	**1.56**	0.08
**Transport**	PYR_IN	**1.32**	0.05	**1.33**	0.02	**1.51**	0.05	**2.75**	0.02
**Transport**	SER_IN	**6.64**	0.09	**6.91**	0.08	**6.73**	0.21	**6.12**	0.11
**Transport**	THR_IN	**2.50**	0.03	**2.52**	0.02	**2.44**	0.10	**1.70**	0.08
**Transport**	TRP_IN	**0.83**	0.01	**0.86**	0.004	**0.79**	0.03	**0.71**	0.03
**Transport**	VAL_IN	**3.58**	0.04	**3.62**	0.03	**3.98**	0.14	**4.47**	0.08
**Calculated from fluxes after fitting:**									
	ATP	**938**	**14.4**	**982**	**12.7**	**911**	**11.6**	**751**	7.8
	NADPH	**42.1**	**9.0**	**38.8**	**5.5**	**30.3**	**7.6**	**29.9**	7.7
	Metabolic Efficiency	**62.7**	**14.4**	**36.4**	**1.2**	**22.1**	**1.7**	**7.0**	0.29
	GDH	**8.5**	**0.23**	**10.2**	**0.28**	**10.1**	**0.40**	**8.5**	0.26

Supp. Table 3: Contribution of individual reactions to ATP generation incl. the factor for multiplication with the respective flux

Reaction	Factor
Glutamate dehydrogenase	1.5
Glyceraldehyde-3-phosphate dehydrogenase	2.5
Hexokinase	-1
Isocitrate dehydrogenase	1.5
α-Ketoglutarate dehydrogenase	2.5
Lactate dehydrogenase	-1
Malic Enzyme	1.5
Phosphofructokinase	(-)1[1]
Pyruvate carboxylase	-1
Pyruvate dehydrogenase	1.5
Pyruvate kinase	1
Succinate dehydrogenase	1

Supp. Table 4: Contribution of individual reactions to NADPH generation incl. the factor for multiplication with the respective flux

Reaction	Factor
Glucose-6-phosphate dehydrogenase	2
Malic Enzyme	1

[1] Due to the inverted direction of the reaction (flux values are negative), the factor is positive, even though the net contribution is negative (consumption).

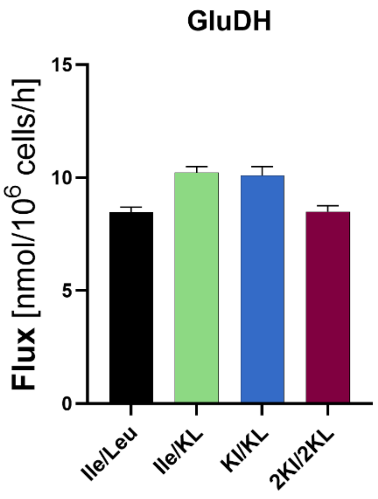

Supp. Figure 1: Glutamate dehydrogenase flux in nmol/10^6 cells/h was calculated within an MFA study on CHOK1 GS in fed-batch using different combinations of branched-chain amino and keto acids in the feed. The MFA software Core*MFA* by Metalytics, Inc. provides a combined flux of reactions forming α-ketoglutarate from L-Glutamate. Considering the fluxes of Aspartate and Alanine transamination, which form Glutamate from α-ketoglutarate, the contribution of Glutamate dehydrogenase was calculated.

2 Perfusion / Metabolomics

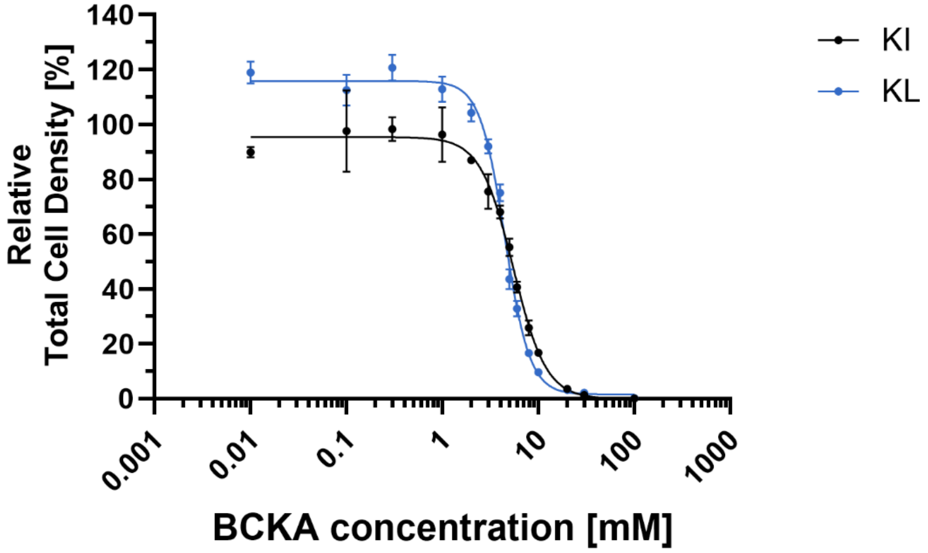

Supp. Figure 2: Growth inhibiting potential of Keto Isoleucine (KI) and Keto Leucine (KL) on CHOK1 GS. Relative Total Cell Density in percent was determined upon BCKA supplementation to determine growth inhibitory effects on CHOK1 GS.

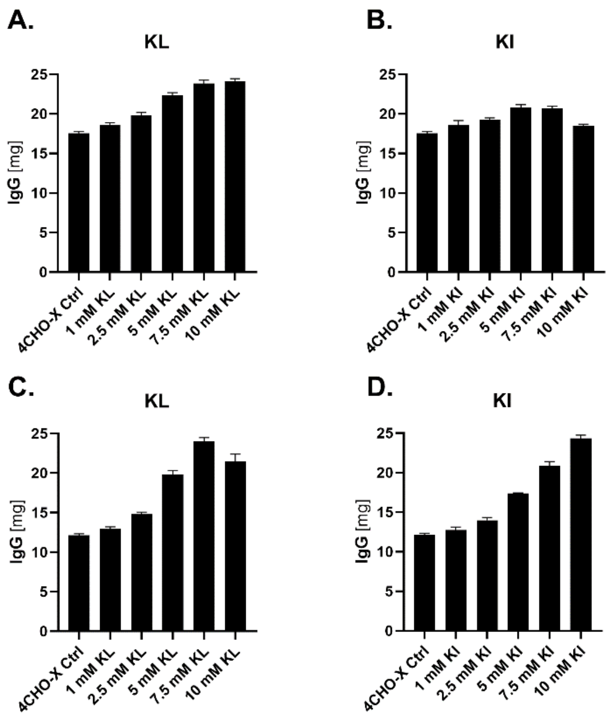

Supp. Figure 3: Total amount of IgG, which was recovered in the harvest of the simulated perfusion experiments with CHOK1 GS and CHO DG44 upon addition of KI and KL in Cellvento® 4CHO-X medium. The bleed was considered waste and was not included in the sum of IgG. A: Addition of KL with CHOK1 GS. B: Addition of KI with CHOK1 GS. C: Addition of KL with CHO DG44. D: Addition of KI with CHO DG44.

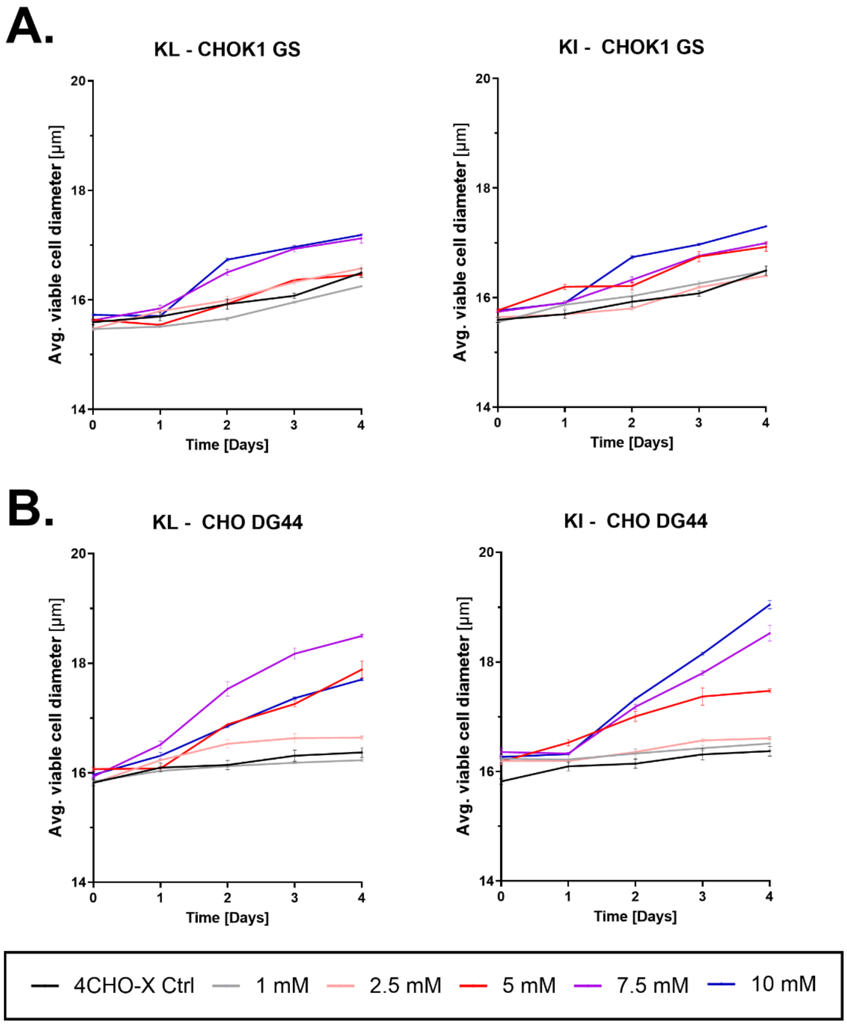

Supp. Figure 4: Average Viable cell diameter [µm] in simulated, steady-state perfusion upon addition of increased Keto Isoleucine (KI) and Keto Leucine (KL) concentrations (n=4). A: CHOK1 GS. B: CHO DG44.

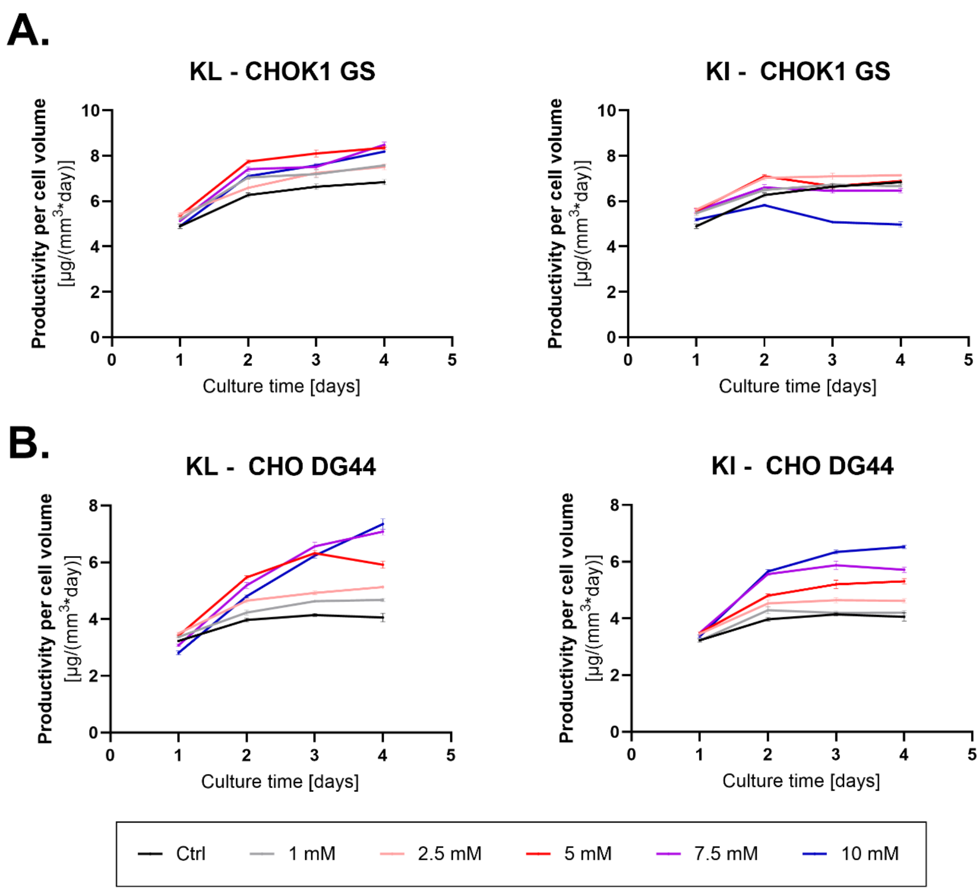

Supp. Figure 5: IgG productivity normalized to the cell volume in µg/(mm^3*day) in steady-state perfusion upon addition of increased Keto Isoleucine (KI) and Keto Leucine (KL) concentrations (n=4). A: CHOK1 GS. B: CHO DG44.

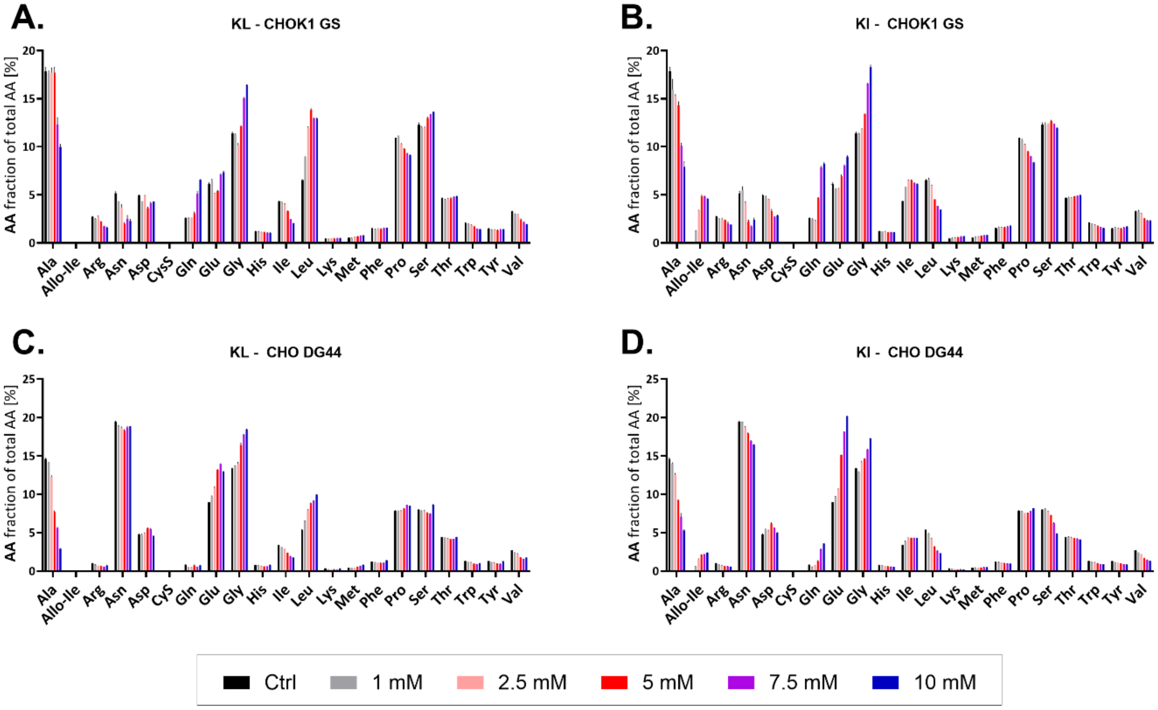

Supp. Figure 6: Intracellular amino acids (represented as percentage of the sum of all amino acids) were determined on day 4 of simulated, steady-state perfusion of CHOK1 GS and CHO DG44 cells upon addition of increasing concentrations of Keto Isoleucine (KI) and Keto Leucine (KL) (n=2). Addition of KL (A) or KI (B) during cultivation of CHOK1 GS and addition of KL (C) or KI (D) during cultivation of CHO DG44.

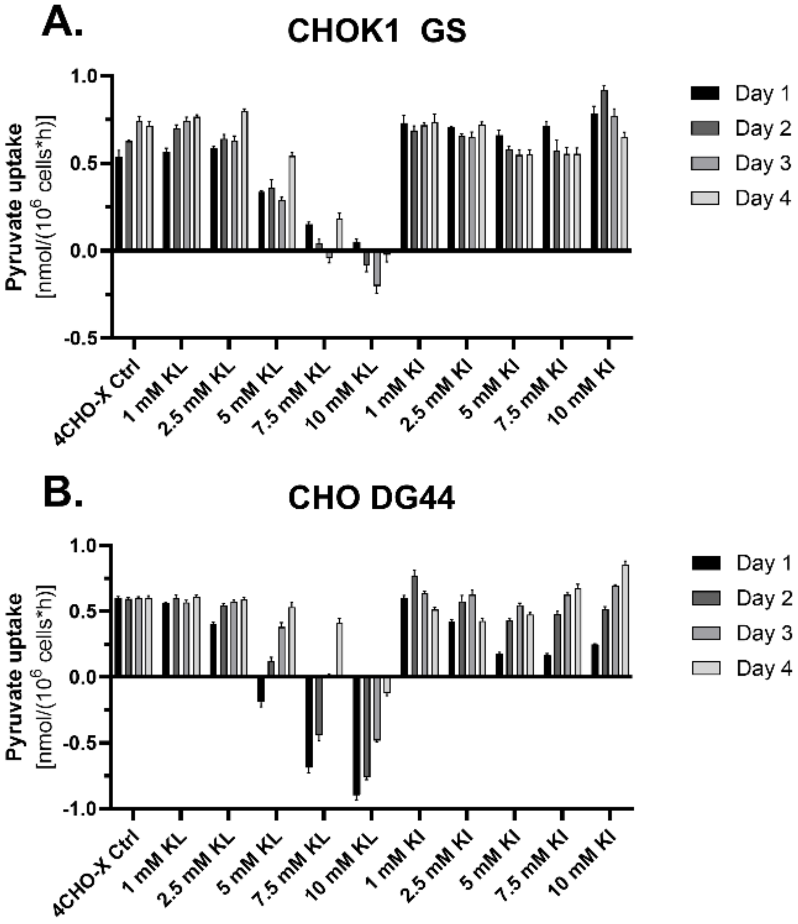

Supp. Figure 7: Specific pyruvate uptake (normalized to the average viable cell concentration) was determined during simulated, steady-state perfusion of CHOK1 GS and CHO DG44 cells upon addition of increasing concentrations of Keto Isoleucine (KI) and Keto Leucine (KL) (n=4). A: CHOK1 GS. B: CHO DG44.

Supp. Table 5: Total number of picked features and explained variances by 1st and 2nd component [%] of the individual PLS regressions

Extra/ Intra	Cell Line	KA	Source Polarity	Total # of features	Explained Variance of Component 1 [%]	Explained Variance of Component 2 [%]
**Extra**	CHOK1 GS	KL	Pos	946	70.2	5.8
**Extra**	CHOK1 GS	KI	Pos	946	67.1	10.8
**Extra**	CHO DG44	KL	Pos	946	73.1	8.9
**Extra**	CHO DG44	KI	Pos	946	61.6	7.7
**Extra**	CHOK1 GS	KL	Neg	123	92.4	1.5
**Extra**	CHOK1 GS	KI	Neg	123	89.3	2.5
**Extra**	CHO DG44	KL	Neg	123	91.9	3.0
**Extra**	CHO DG44	KI	Neg	123	84.3	6.1
**Intra**	CHOK1 GS	KL	Pos	942	84.2	4.1
**Intra**	CHOK1 GS	KI	Pos	942	57	29
**Intra**	CHO DG44	KL	Pos	1463	74.2	10.3
**Intra**	CHO DG44	KI	Pos	1463	77.2	4.4
**Intra**	CHOK1 GS	KL	Neg	315	89.6	5.7
**Intra**	CHOK1 GS	KI	Neg	315	77.2	12.5
**Intra**	CHO DG44	KL	Neg	391	84.4	6.5
**Intra**	CHO DG44	KI	Neg	391	85.7	2.4

Supp. Table 6: Overview of compounds identified in the metabolomics study of a simulated, steady-state perfusion process with CHOK1 GS and CHO DG44. The list contains abbreviations, a name or description of the molecule, IUPAC names, tiers, retention time (RT), most abundant ion and respective m/z ratio, and is limited to molecules, which are mentioned in the manuscript.

#	Abbreviation	Name / Description	Sum formula	IUPAC	Tier	RT [min]	m/z ratio	Ion
**1**	KI	keto isoleucine	C6H10O3	3-methyl-2-oxopentanoic acid	1/1'	6.5	129.05572	[M-H]-
**2**	KL	keto leucine	C6H10O3	4-methyl-2-oxopentanoic acid	1/1'	6.8	129.0557	[M-H]-
**3**	KV	keto valine	C5H8O3	3-methyl-2-oxobutanoic acid	1/1'	4.2	115.04002	[M-H]-
**4**	CP	thiazolidine formed from pyruvate and cysteine	C6H9NO4S	2-methyl-1,3-thiazolidine-2,4-dicarboxylic acid	1	1.5	192.03239	[M+H]+
**5**	CKG	thiazolidine formed from α-ketoglutaric acid and cysteine	C8H11NO6S	3-[2,4-bis(dihydroxymethyl)-1,3-thiazolidin-2-yl]propanoic acid	1	1.8	250.03797	[M+H]+
**6**	CKL	thiazolidine formed from keto leucine and cysteine	C9H15NO4S	2-(2-methylpropyl)-1,3-thiazolidine-2,4-dicarboxylic acid	1	5.4	234.07938	[M+H]+
**7**	CKI	thiazolidine formed from keto isoleucine and cysteine	C9H15NO4S	2-(butan-2-yl)-1,3-thiazolidine-2,4-dicarboxylic acid	1	5.7	234.07945	[M+H]+
**8**	Th-KI	1-hydroxy-2-methylbutyl thiamine	C17H27N4O2S	3-[(4-amino-2-methylpyrimidin-5-yl)methyl]-2-(1-hydroxy-2-methylbutyl)-5-(2-hydroxyethyl)-4-methyl-1,3-thiazol-3-ium	1	7.2/7.5 (2 peaks)	351.18502	[M]+
**9**	Th-KL	1-hydroxy-3-methylbutyl thiamine	C17H27N4O2S	3-[(4-amino-2-methylpyrimidin-5-yl)methyl]-2-(1-hydroxy-3-methylbutyl)-5-(2-hydroxyethyl)-4-methyl-1,3-thiazol-3-ium	1	7.3	351.18501	[M]+
**10**	Th-KV	1-hydroxy-2-methylpropyl thiamine	C16H25N4O2S	3-[(4-amino-2-methylpyrimidin-5-yl)methyl]-2-(1-hydroxy-2-methylpropyl)-5-(2-hydroxyethyl)-4-methyl-1,3-thiazol-3-ium	1/1'	6.1	337.16965	[M]+
**11**	-	Thiamine	C12H17N4OS	3-[(4-amino-2-methylpyrimidin-5-yl)methyl]-5-(2-hydroxyethyl)-4-methyl-1,3-thiazol-3-ium	1	2.7	265.11181	[M]+
**12**	-	Putative CKL degradation product	C8H13NO2S	2-isobutyl-4,5-dihydrothiazole-4-carboxylic acid	3	9.1	188.07389	[M]+
**13**	-	Putative Pyr+KL cross-aldol reaction product	C9H14O6	2-hydroxy-2-(2-methylpropyl)-4-oxopentanedioic acid	3	7.7	217.07169	[M-H]-
**14**	-	Riboflavin	C17H20N4O6	7,8-dimethyl-10-[(2S,3S,4R)-2,3,4,5-tetrahydroxypentyl]-2H,3H,4H,10H-benzo[g]pteridine-2,4-dione	1'	8.6	399.12782	[M+H]+
**15**	γ-Glu-Leu	γ-glutamyl leucine	C11H20N2O5	(2S)-2-[(4S)-4-amino-4-carboxybutanamido]-4-methylpentanoic acid	1	7.5	261.14445	[M+H]+
**16**	γ-Glu-Ile	γ-glutamyl isoleucine	C11H20N2O5	(2S,3S)-2-[(4S)-4-amino-4-carboxybutanamido]-3-methylpentanoic acid	1	7.2	261.14448	[M+H]+
**17**	NAc-Leu	N-acetyl leucine	C8H15NO3	(2S)-2-acetamido-4-methylpentanoic acid	1	8.9	172.09792	[M-H]-
**18**	NAc-Ile	N-acetyl isoleucine	C8H15NO3	(2S,3S)-2-acetamido-3-methylpentanoic acid	1/1'	8.7	172.0979	[M-H]-
**19**	HI	α-hydroxy acid of isoleucine	C6H12O3	2-hydroxy-3-methylpentanoic acid	1/1'	8.7	131.07134	[M-H]-

#	Abbreviation	Name / Description	Sum formula	IUPAC	Tier	RT [min]	m/z ratio	Ion
**20**	HL	α-hydroxy acid of leucine	C6H12O3	2-hydroxy-4-methylpentanoic acid	1/1'	8.8	131.07137	[M-H]-
**21**	HV	α-hydroxy acid of valine	C5H10O3	2-hydroxy-3-methylbutanoic acid	1	6.7	117.05569	[M-H]-
**22**	Gly-CKL	thiazolidine formed from keto leucine and cysteine-glycine	C11H18N2O5S	2-(butan-2-yl)-4-[(carboxymethyl)carbamoyl]-1,3-thiazolidine-2-carboxylic acid	1'	8.2/9.0 (2 peaks)	291.101	[M+H]+
**23**	Gly-CKI	thiazolidine formed from keto isoleucine and cysteine-glycine	C11H18N2O5S	4-[(carboxymethyl)carbamoyl]-2-(2-methylpropyl)-1,3-thiazolidine-2-carboxylic acid	1/1'	8.82	291.10079	[M+H]+
**24**	CKV	thiazolidine formed from keto valine and cysteine	C8H13NO4S	2-isopropyl-1,3-thiazolidine-2,4-dicarboxylic acid	1/1'	3.3	220.06375	[M+H]+
**25**	ThP-KI	1-hydroxy-2-methylbutyl thiamine monophosphate	C17H28N4O5PS	3-[(4-amino-2-methylpyrimidin-5-yl)methyl]-2-(1-hydroxy-2-methylbutyl)-4-methyl-5-[2-(phosphonooxy)ethyl]-1,3-thiazol-3-ium	3	6.6	431.15145	[M]+
**26**	ThPP-KI	1-hydroxy-2-methylbutyl thiamine diphosphate	C17H29N4O8P2S	3-[(4-amino-2-methylpyrimidin-5-yl)methyl]-5-(2-{[hydroxy(phosphonooxy)phosphoryl]oxy}ethyl)-2-(1-hydroxy-2-methylbutyl)-4-methyl-1,3-thiazol-3-ium	3	6.3	511.11764	[M]+
**27**	ThP-KL	1-hydroxy-3-methylbutyl thiamine monophosphate	C17H28N4O5PS	3-[(4-amino-2-methylpyrimidin-5-yl)methyl]-2-(1-hydroxy-3-methylbutyl)-4-methyl-5-[2-(phosphonooxy)ethyl]-1,3-thiazol-3-ium	3	6.6	431.1515	[M]+
**28**	ThPP-KL	1-hydroxy-3-methylbutyl thiamine diphosphate	C17H29N4O8P2S	3-[(4-amino-2-methylpyrimidin-5-yl)methyl]-5-(2-{[hydroxy(phosphonooxy)phosphoryl]oxy}ethyl)-2-(1-hydroxy-3-methylbutyl)-4-methyl-1,3-thiazol-3-ium	3	6.3	511.11764	[M]+
**29**	ThP-KV	1-hydroxy-2-methylpropyl thiamine monophosphate	C16H26N4O5PS	3-[(4-amino-2-methylpyrimidin-5-yl)methyl]-2-(1-hydroxy-2-methylpropyl)-4-methyl-5-[2-(phosphonooxy)ethyl]-1,3-thiazol-3-ium	3	5.5	417.13562	[M]+
**30**	ThPP-KV	1-hydroxy-2-methylpropyl thiamine diphosphate	C16H27N4O8P2S	3-[(4-amino-2-methylpyrimidin-5-yl)methyl]-5-(2-{[hydroxy(phosphonooxy)phosphoryl]oxy}ethyl)-2-(1-hydroxy-2-methylpropyl)-4-methyl-1,3-thiazol-3-ium	3	5.2	497.10244	[M]+
**31**	Th-KG	3-carboxy-1-hydroxypropyl thiamine	C16H23N4O4S	3-[(4-amino-2-methylpyrimidin-5-yl)methyl]-2-(3-carboxy-1-hydroxypropyl)-5-(2-hydroxyethyl)-4-methyl-1,3-thiazol-3-ium	3	4.7	367.14345	[M]+
**32**	Th-Pyr	1-hydroxyethyl thiamine	C14H21N4O2S	3-[(4-amino-2-methylpyrimidin-5-yl)methyl]-2-(1-hydroxyethyl)-5-(2-hydroxyethyl)-4-methyl-1,3-thiazol-3-ium	1	4.2	309.13797	[M]+
**33**	ThP-Pyr	1-hydroxyethyl thiamine monophosphate	C14H22N4O5PS	3-[(4-amino-2-methylpyrimidin-5-yl)methyl]-2-(1-hydroxyethyl)-4-methyl-5-[2-(phosphonooxy)ethyl]-1,3-thiazol-3-ium	3	2.1	389.10433	[M]+
**34**	ThPP-Pyr	1-hydroxyethyl thiamine diphosphate	C14H23N4O8P2S	3-[(4-amino-2-methylpyrimidin-5-yl)methyl]-5-(2-{[hydroxy(phosphonooxy)phosphoryl]oxy}ethyl)-2-(1-hydroxyethyl)-4-methyl-1,3-thiazol-3-ium	3	1.8	469.07081	[M]+
**35**	dcKL/dcKI	decarboxylation products of keto leucine and keto isoleucine	C5H10O2	3-methylbutanoic acid	1'	9.3	101.0608	[M-H]-
**36**	-	dcKI/dcKL-glucuronide	C11H18O8	(2S,3S,4S,5R,6S)-3,4,5-trihydroxy-6-[(3-methylbutanoyl)oxy]oxane-2-carboxylic acid / (2S,3S,4S,5R,6S)-3,4,5-trihydroxy-6-{[(2S)-2-methylbutanoyl]oxy}oxane-2-carboxylic acid	1/1*	7.5	277.09296	[M-H]-

#	Abbreviation	Name / Description	Sum formula	IUPAC	Tier	RT [min]	m/z ratio	Ion
**37**	HMB	3-hydroxyisovaleric acid	C5H10O3	3-hydroxy-3-methylbutanoic acid	1/1'	5.9	117.05574	[M-H]-
**38**	HMG	3-hydroxy-3-methylglutaric acid	C6H10O5	3,5,5-trihydroxy-3-methylpentanoic acid	1	4.5	161.0456	[M-H]-
**39**	HMH	(3S)-3-hydroxy-5-methylhexanoic acid	C7H14O3	(3S)-3-hydroxy-5-methylhexanoic acid	1/1'	9.3	145.087	[M-H]-
**40**	H-Trp	α-hydroxy acid of tryptophan	C11H11NO3	2-hydroxy-3-(1H-indol-3-yl)propanoic acid	1	8.9	204.0666	[M-H]-
**41**	H-Phe	α-hydroxy acid of phenylalanine	C9H10O3	2-hydroxy-3-phenylpropanoic acid	1	8.9	165.05572	[M-H]-
**42**	H-Met	α-hydroxy acid of methionine	C5H10O3S	2-hydroxy-4-(methylsulfanyl)butanoic acid	1'	6.4	151.04226	[M+H]+
**43**	H-Tyr	α-hydroxy acid of tyrosine	C9H10O4	2-hydroxy-3-(4-hydroxyphenyl)propanoic acid	1	6.9	181.05059	[M-H]-
**44**	H-Glu	α-hydroxy acid of glutamate	C5H8O5	2-hydroxypentanedioic acid	1/1'	2.1	147.02991	[M-H]-
**45**	NAc-Tyr	N-acetyl tyrosine	C11H13NO4	(2S)-2-acetamido-3-(4-hydroxyphenyl)propanoic acid	1'	7	222.07722	[M-H]-
**46**	NAc-Gln	N-acetyl glutamine	C7H12N2O4	(2S)-4-carbamoyl-2-acetamidobutanoic acid	1/1'	1.9	189.08699	[M+H]+
**47**	N6-Ac-Lys	N6-acetyl lysine	C8H16N2O3	(2S)-2-amino-6-acetamidohexanoic acid	1	2.1	189.12344	[M+H]+
**48**	NAc-Met	N-acetyl methionine	C7H13NO3S	(2S)-2-acetamido-4-(methylsulfanyl)butanoic acid	1/1'	6.9	192.06889	[M+H]+
**49**	NAc-Arg	N-acetyl arginine	C8H16N4O3	(2S)-5-carbamimidamido-2-acetamidopentanoic acid	1/1'	2.5	217.12948	[M+H]+
**50**	NAc-Ser	N-acetyl serine	C5H9NO4	(2S)-2-acetamido-3-hydroxypropanoic acid	1/1'	1.7	146.0459	[M-H]-
**51**	NAc-Trp	N-acetyl tryptophan	C13H14N2O3	(2S)-2-acetamido-3-(1H-indol-3-yl)propanoic acid	1	8.9	247.10778	[M+H]+
**52**	NAc-Ala	N-acetyl alanine	C5H9NO3	(2S)-2-acetamidopropanoic acid	1'	3.4	130.05105	[M-H]-
**53**	γ-Glu-Tyr	γ-glutamyl tyrosine	C14H18N2O6	(2S)-2-amino-4-{[(1S)-1-carboxy-2-(4-hydroxyphenyl)ethyl]carbamoyl}butanoic acid	1/1'	5.8	311.12392	[M+H]+
**54**	γ-Glu-Phe	γ-glutamyl phenylalanine	C14H18N2O5	(2S)-2-amino-4-{[(1S)-1-carboxy-2-phenylethyl]carbamoyl}butanoic acid	1	7.7	295.12885	[M+H]+
**55**	γ-Glu-Met	γ-glutamyl methionine	C10H18N2O5S	(2S)-2-amino-4-{[(1S)-1-carboxy-3-(methylsulfanyl)propyl]carbamoyl}butanoic acid	3	5.4	279.10089	[M+H]+
**56**	γ-Glu-Trp	γ-glutamyl tryptophan	C16H19N3O5	(2S)-2-amino-4-{[(1S)-1-carboxy-2-(1H-indol-3-yl)ethyl]carbamoyl}butanoic acid	3	8.3	334.13984	[M+H]+
**57**	γ-Glu-Thr	γ-glutamyl threonine	C9H16N2O6	(2S)-2-amino-4-{[(1S,2R)-1-carboxy-2-hydroxypropyl]carbamoyl}butanoic acid	3	1.6	249.10797	[M+H]+
**58**	γ-Glu-Arg	γ-glutamyl arginine	C11H21N5O5	(2S)-2-[(4S)-4-amino-4-carboxybutanamido]-5-carbamimidamidopentanoic acid	1	1.6	304.16162	[M+H]+
**59**	γ-Glu-Ser	γ-glutamyl serine	C8H14N2O6	(2S)-2-amino-4-{[(1S)-1-carboxy-2-hydroxyethyl]carbamoyl}butanoic acid	1'	1.3	235.09256	[M+H]+
**60**	γ-Glu-Glu	γ-glutamyl glutamic acid	C10H16N2O7	(2S)-2-[(4S)-4-amino-4-carboxybutanamido]pentanedioic acid	3	3.8	275.08879	[M-H]-
**61**	γ-Glu-Gln	γ-glutamyl glutamine	C10H17N3O6	(2S)-2-[(4S)-4-amino-4-carboxybutanamido]-4-carbamoylbutanoic acid	1'	1.5	276.11902	[M+H]+
**62**	γ-Glu-Ala	γ-glutamyl alanine	C8H14N2O5	(2S)-2-amino-4-{[(1S)-1-carboxyethyl]carbamoyl}butanoic acid	1/1'	1.8	219.09749	[M+H]+
**63**	γ-Glu-Lys	γ-glutamyl lysine	C11H21N3O5	(2S)-6-amino-2-[(4S)-4-amino-4-carboxybutanamido]hexanoic acid	3	1.8	187.10769	[M+H-C3H7NO2]+

#	Abbreviation	Name / Description	Sum formula	IUPAC	Tier	RT [min]	m/z ratio	Ion
**64**	γ-Glu-Cys-Gly (GSH)	Glutathione (reduced)	C10H17N3O6S	(2S)-2-amino-4-{[(1R)-1-[(carboxymethyl)carbamoyl]-2-sulfanylethyl]carbamoyl}butanoic acid	1	2.2	308.09102	[M+H]+
**65**	GSSG	Glutathione (oxidized)	C20H32N6O12S2	2-amino-5-[[1-[[[2-[(4-amino-4-carboxy-butanoyl)amino]-3-(carboxymethylamino)-3-oxo-propyl]disulfanyl]methyl]-2-(carboxymethylamino)-2-oxo-ethyl]amino]-5-oxo-pentanoic acid	1	3.8	613.1592	[M+H]+
**66**	NAc-Cad	N-acetyl cadaverine	C7H16N2O	N-(5-aminopentyl)acetamide	1/1'	3.5	145.13336	[M+H]+
**67**	dCyt	2'-deoxycytidine	C9H13N3O4	4-amino-1-[(2R,4S,5R)-4-hydroxy-5-(hydroxymethyl)oxolan-2-yl]-1,2-dihydropyrimidin-2-one	1	4.3	228.09785	[M+H]+
**68**	MSA	methylsuccinic acid	C5H8O4	2-methylbutanedioic acid	1/1'	6	131.03497	[M-H]-
**69**	EMA	ethylmalonic acid	C5H8O4	2-ethylpropanedioic acid	1/1'	5.1	87.04508	[M-H-CO2]-
**70**	-	N8-acetyl-spermidine	C9H21N3O	N-{4-[(3-aminopropyl)amino]butyl}acetamide	1/1'	1.7	188.17573	[M+H]+
**71**	KG	α-ketoglutaric acid	C5H6O5	2-oxopentanedioic acid	1	1.7	145.01425	[M-H]-

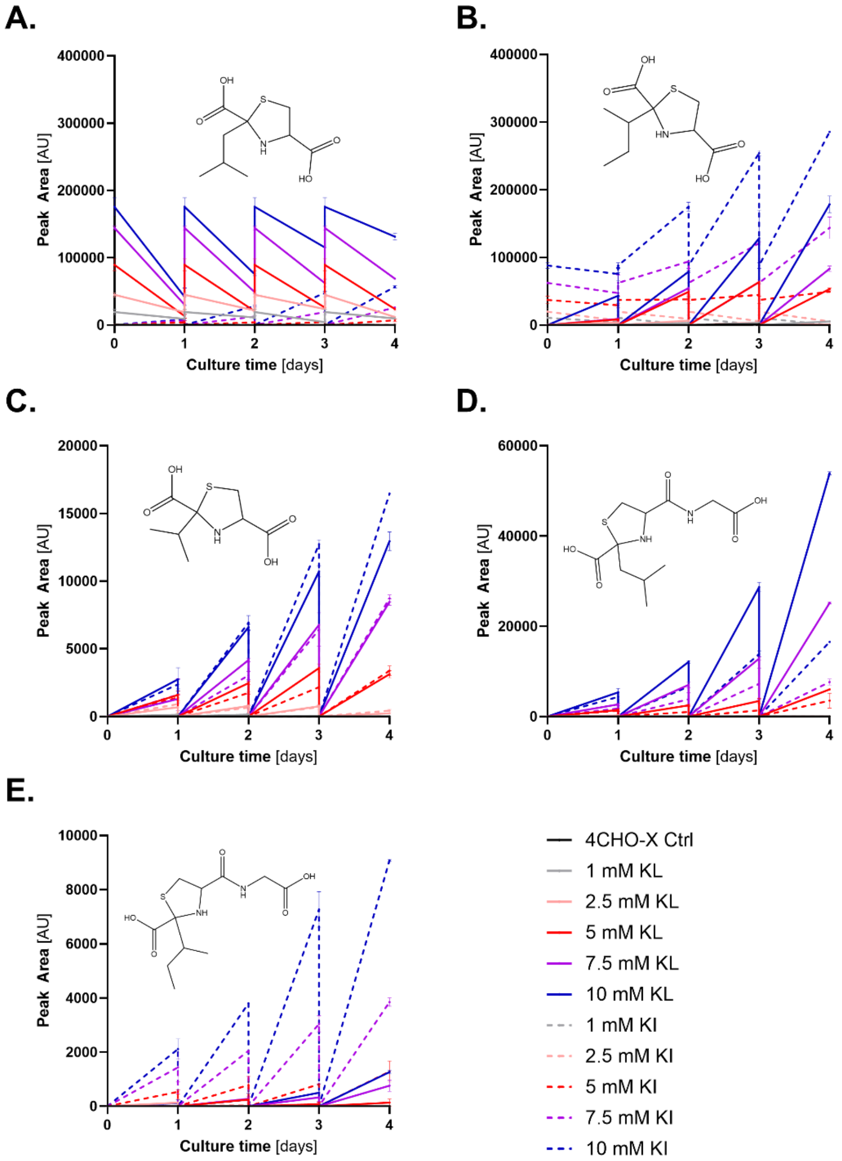

Supp. Figure 8: Abundance profiles of thiazolidine derivatives formed from the keto acids of valine, leucine or isoleucine with cysteine or cysteine-glycine, which were identified in the (spent) media of simulated perfusion processes with CHOK1 GS (shown) and CHO DG44 (not shown) (n=2). A: 2-(2-methylpropyl)-1,3-thiazolidine-2,4-dicarboxylic acid (CKL), the thiazolidine formed from cysteine and keto leucine. B: 2-(sec-butyl)-1,3-thiazolidine-2,4-dicarboxylic acid, the thiazolidine formed from cysteine and keto isoleucine (CKI). C: 2-isopropyl-1,3-thiazolidine-2,4-dicarboxylic acid, the thiazolidine formed from cysteine and keto valine (CKV). D: 4-(carboxymethylcarbamoyl)-2-(2-methylpropyl)-1,3-thiazolidine-2-carboxylic acid, the thiazolidine formed from Cys-Gly and keto leucine (Gly-CKL). E: 4-(carboxymethylcarbamoyl)-2-(sec-butyl)-1,3-thiazolidine-2-carboxylic acid, the thiazolidine formed from Cys-Gly and keto isoleucine (Gly-CKI).

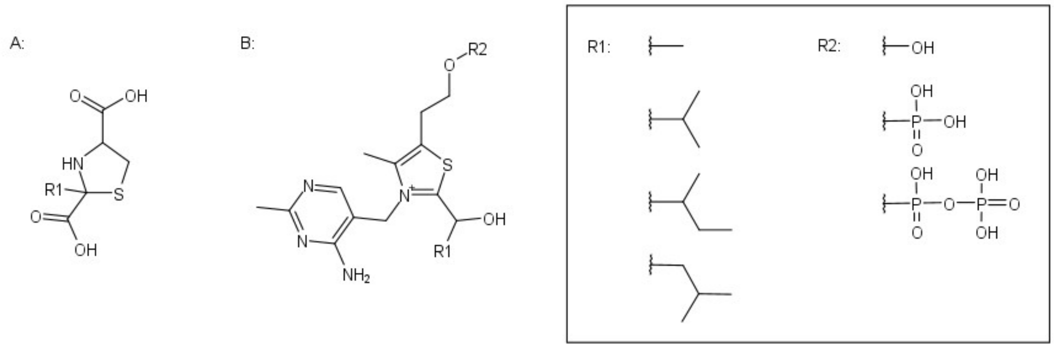

Supp. Figure 9: Thiazolidine and thiamine (mono-/diphosphate) derivatives of pyruvate and the keto acids of valine, leucine and isoleucine, which were identified extra- or intracellularly in the metabolome of CHOK1 GS and CHO DG44. A: Thiazolidines formed from cysteine and an α-keto acid. B: Thiamine (mono-/diphosphate) derivatives formed from thiamine and an α-keto acid.

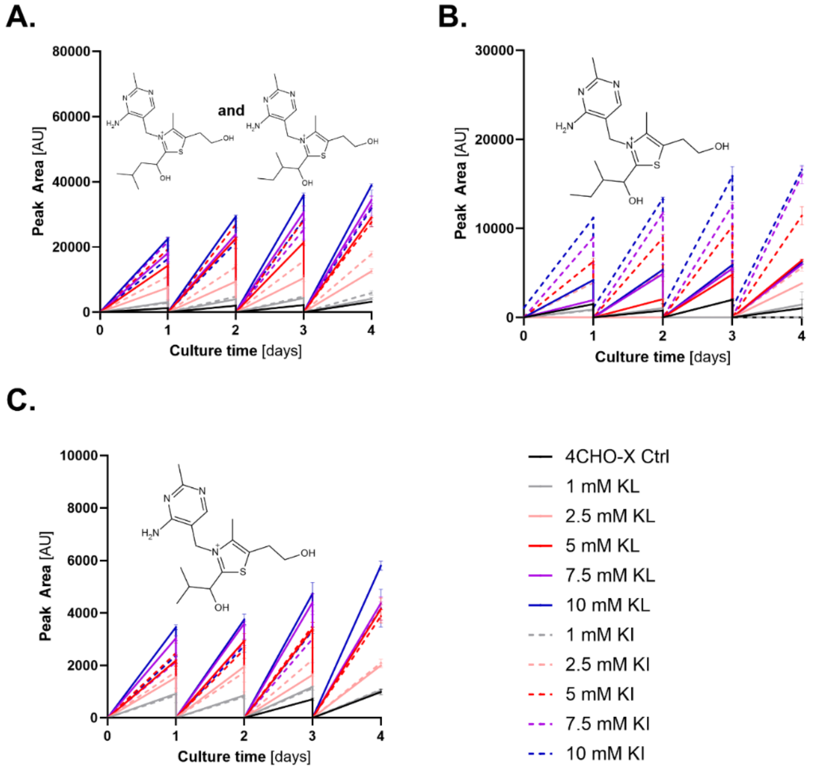

Supp. Figure 10: Abundance profiles of hydroxyalkyl thiamines, which were identified in the (spent) media of simulated perfusion processes with CHOK1 GS (shown) and CHO DG44 (not shown) (n=2). A: Due to co-elution, this feature is a sum parameter of 1-hydroxy-3-methylbutyl thiamine (Th-KL) and one isomer of 1-hydroxy-2-methylbutyl thiamine (Th-KI). B: Second isomer of 1-hydroxy-2-methylbutyl thiamine (Th-KI). C: 1-hydroxy-2-methylpropyl thiamine (Th-KV).

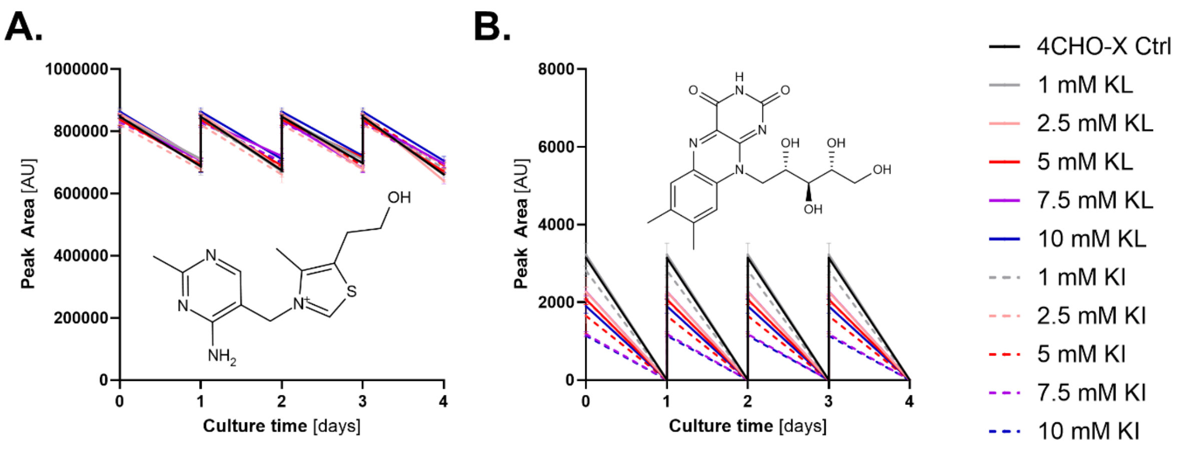

Supp. Figure 11: Abundance profiles (extracellular) of thiamine and riboflavin, which were part of media components in simulated perfusion processes with CHOK1 GS (shown) and CHO DG44 (not shown) (n=2). A: Thiamine. B: Riboflavin.

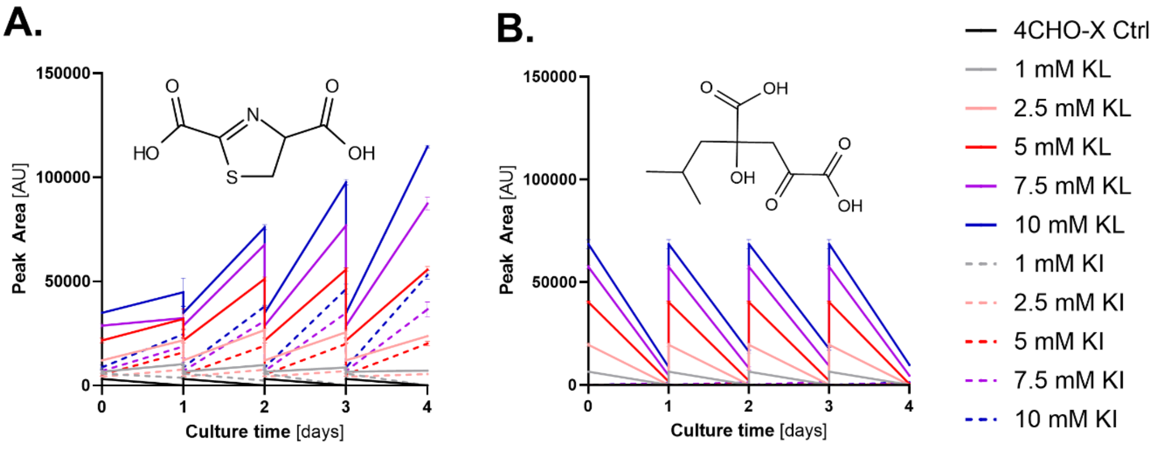

Supp. Figure 12: Abundance profiles of putative keto leucine interaction products, which were identified in the (spent) media of simulated perfusion processes with CHOK1 GS (shown) and CHO DG44 (not shown) (n=2). A: 2-isobutyl-4,5-dihydrothiazole-4-carboxylic acid, the putative degradation product of the thiazolidine formed from keto leucine and cysteine. B: 2-hydroxy-2-(2-methylpropyl)-4-oxopentanedioic acid, the putative cross-aldol reaction product formed from keto leucine and pyruvate.

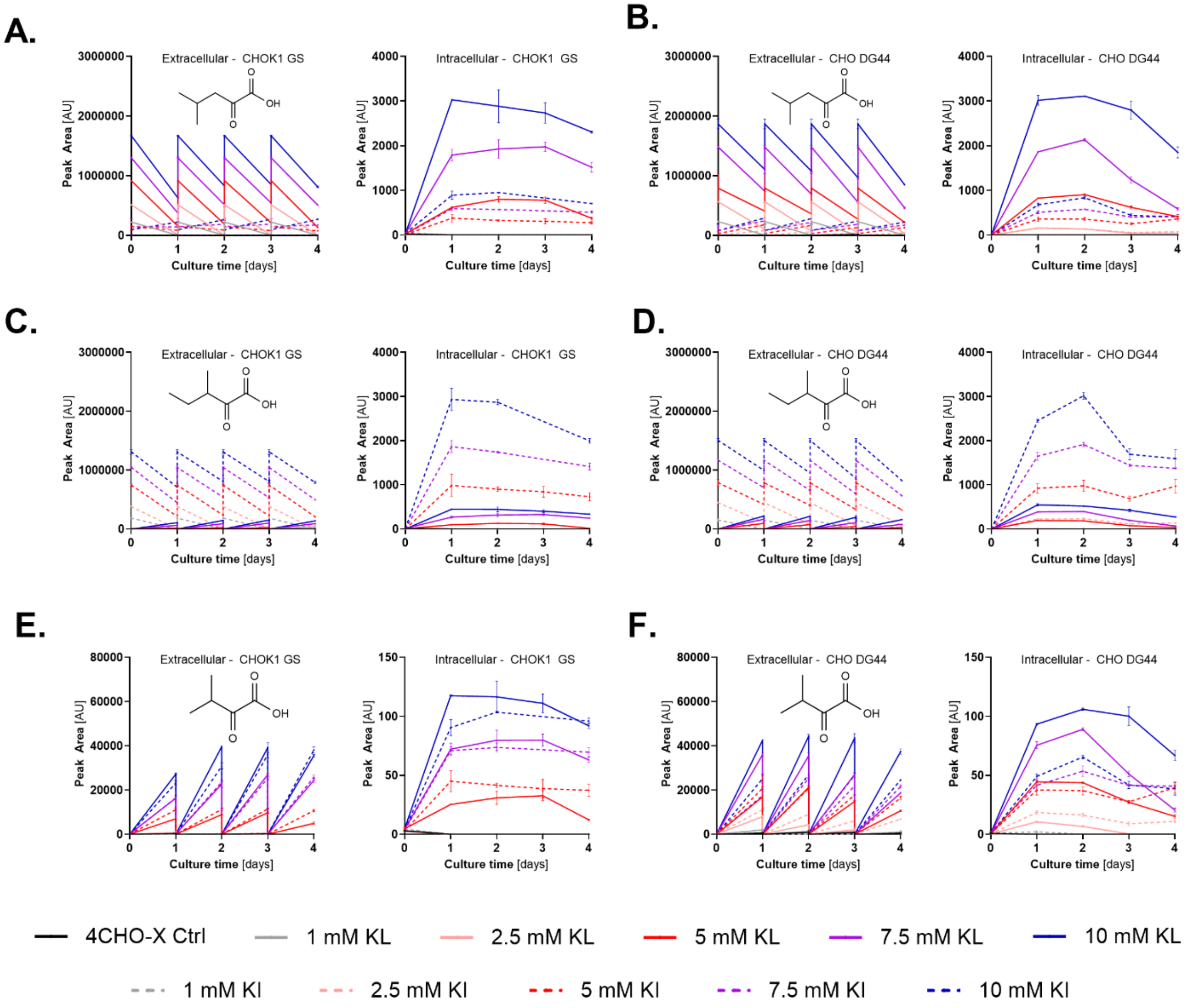

Supp. Figure 13: Abundance profiles of BCKAs in simulated perfusion with either CHOK1 GS or CHO DG44. Intracellular values were normalized to the total protein content of the extracted pellet (n=2). A: Keto leucine (KL) CHOK1 GS. B: KL with CHO DG44. C: Keto isoleucine (KI) with CHOK1 GS. D: KI with CHO DG44. E: Keto valine (KV) with CHOK1 GS. F: KV with CHO DG44.

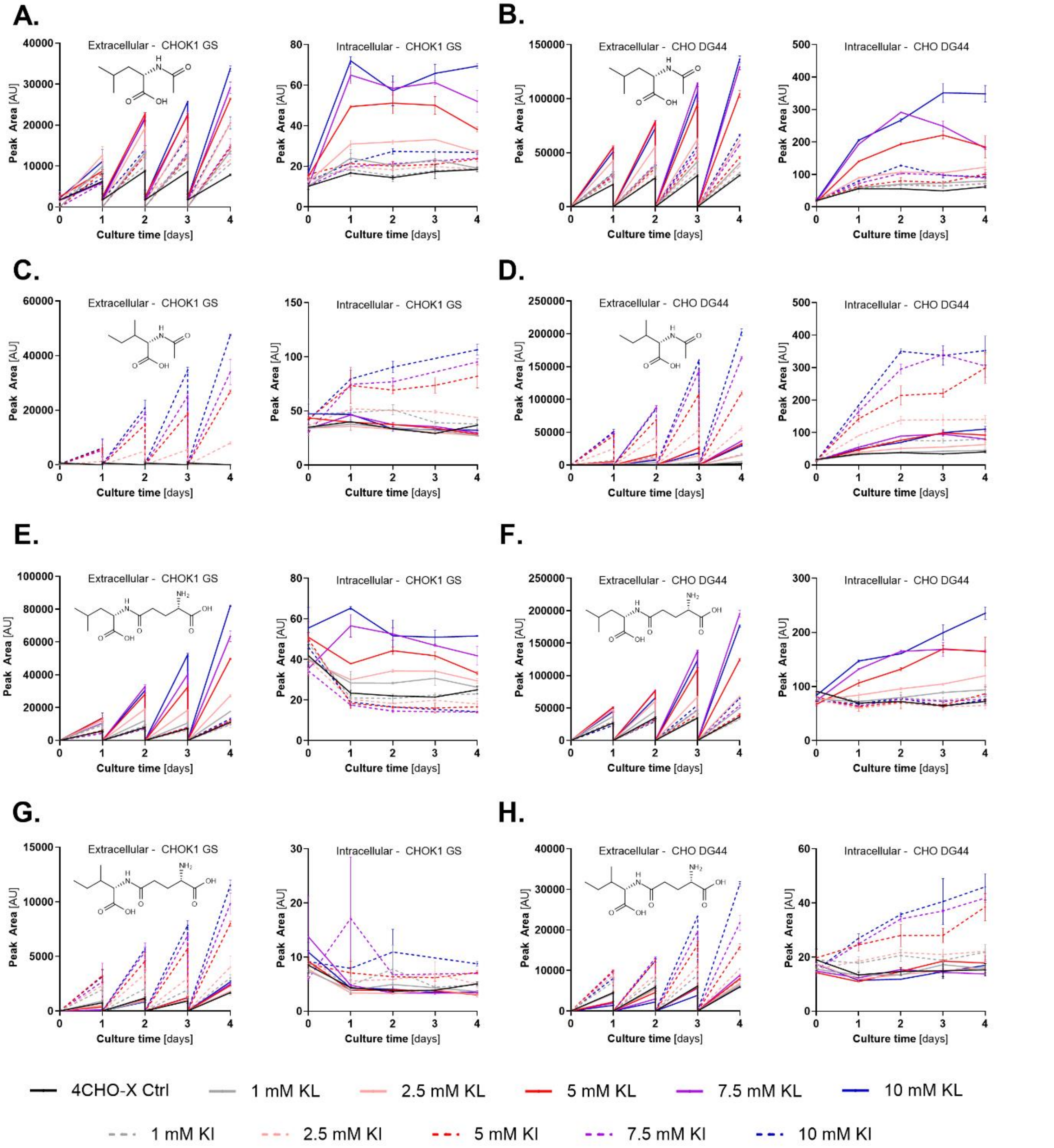

Supp. Figure 14: Intra- and extracellular abundance profiles of N-acetylated, and γ-glutamyl derivatives of leucine (Leu) and isoleucine (Ile), which were impacted by supplementation of keto isoleucine and keto leucine in simulated perfusion with CHOK1 GS and CHO DG44. Intracellular values were normalized to the total protein content of the extracted pellet (n=2). A: N-acetyl leucine (NAc-Leu) with CHOK1 GS. B: NAc-Leu with CHO DG44. C: N-acetyl isoleucine (NAc-Ile) with CHOK1 GS. D: NAc-Ile with CHO DG44. E: γ-glutamyl leucine (γ-Glu-Leu) with CHOK1 GS. F: γ-Glu-Leu with CHO DG44. G: γ-glutamyl isoleucine (γ-Glu-Ile) with CHOK1 GS. H: γ-Glu-Ile with CHO DG44.

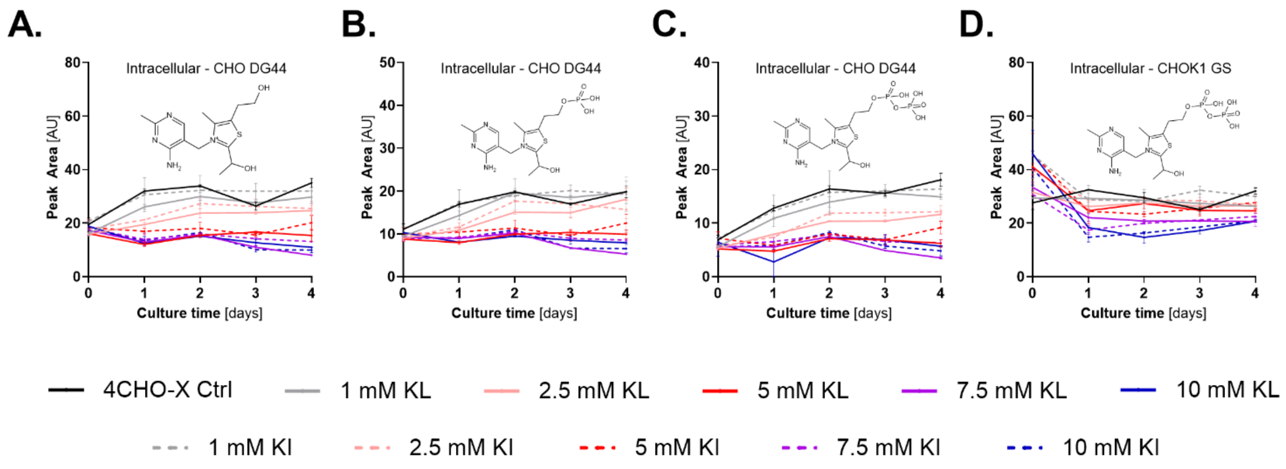

Supp. Figure 15: Abundance profiles of 1-hydroxyethyl thiamine (Th-Pyr) and its mono- (ThP-Pyr) and diphosphate (ThPP-Pyr) identified in the intracellular content of CHO DG44 and CHOK1 GS during a simulated perfusion process. This molecule is the interaction product of thiamine (mono-/diphosphate) with pyruvate. Intracellular values were normalized to the total protein content of the extracted pellet (n=2). A: Th-Pyr with CHO DG44. B: ThP-Pyr with CHO DG44. C: ThPP-Pyr with CHO DG44. D: ThPP-Pyr with CHOK1 GS.

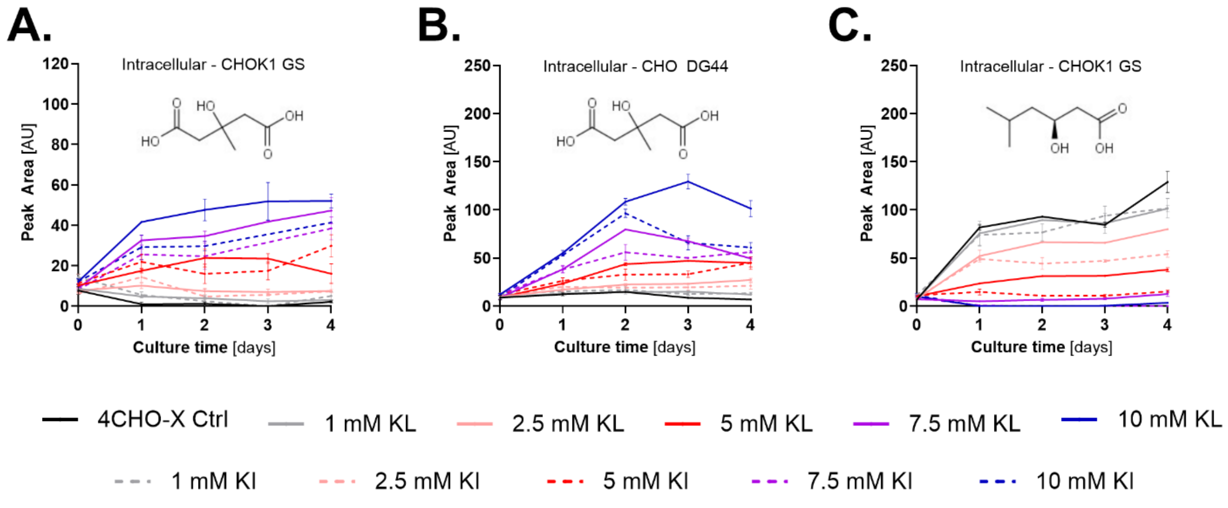

Supp. Figure 16: Abundance profiles of 3-hydroxy-3-methylglutaric acid (HMG) and (3S)-3-hydroxy-5-methylhexanoic acid (HMH), which were identified in the intracellular content of CHOK1 GS, CHO DG44 or both in simulated perfusion. Intracellular values were normalized to the total protein content of the extracted pellet (n=2). A: HMG in CHOK1 GS. B: HMG in CHO DG44. C: HMH in CHOK1 GS.

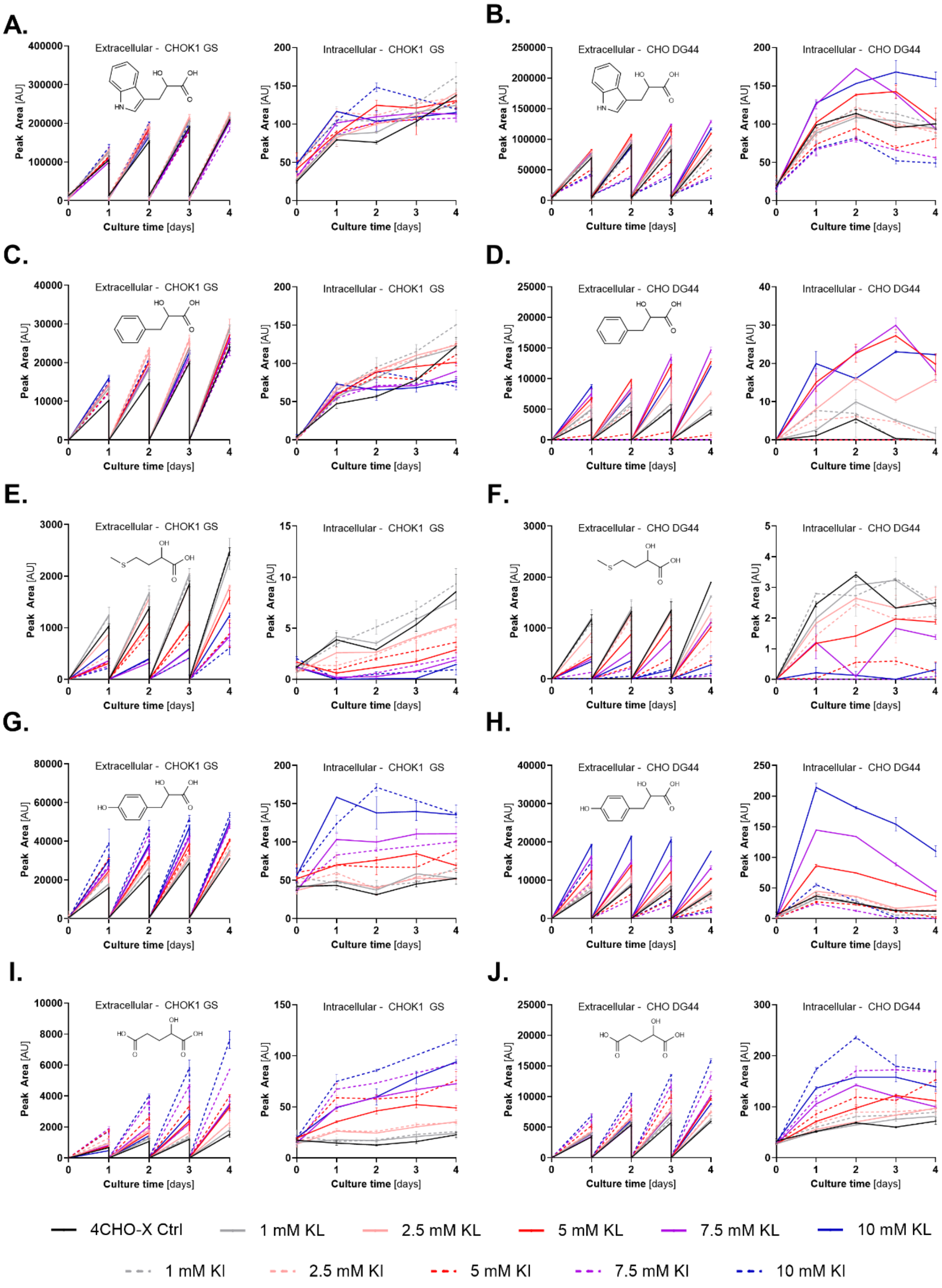

Supp. Figure 17: Intra- and extracellular abundance profiles of α-hydroxy acids derived from tryptophan (H-Met), phenylalanine (H-Phe), methionine (H-Met), tyrosine (H-Tyr) and glutamate (H-Glu), which were impacted by supplementation of keto isoleucine and keto leucine in simulated perfusion with CHOK1 GS and CHO DG44. Intracellular values were normalized to the total protein content of the extracted pellet (n=2). A: H-Trp with CHOK1 GS. B: H-Trp with CHO DG44. C: H-Phe with CHOK1 GS. D: H-Phe with CHO DG44. E: H-Met with CHOK1 GS. F: H-Met with CHO DG44. G: H-Tyr with CHOK1 GS. H: H-Tyr with CHO DG44. I: H-Glu with CHOK1 GS. J: H-Glu with CHO DG44.

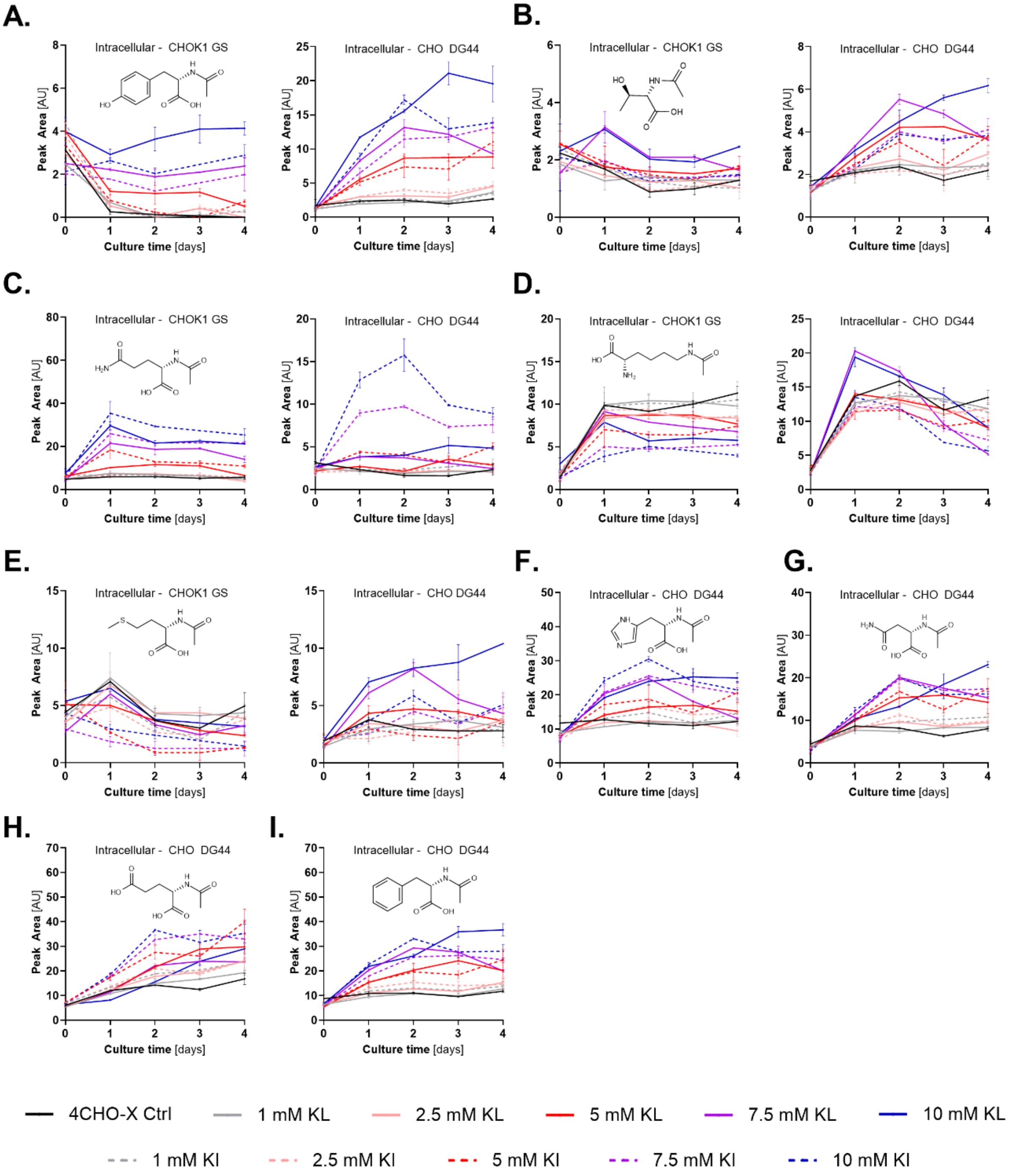

Supp. Figure 18: Abundance profiles of N-acetylated amino acids (NAc-AA), identified in the intracellular content of CHO DG44 and CHOK1 GS during simulated perfusion. Intracellular values were normalized to the total protein content of the extracted pellet (n=2). A: NAc-Tyr in CHOK1 GS and CHO DG44. B: NAc-Thr in CHOK1 GS and CHO DG44. C: NAc-Gln in CHOK1 GS and CHO DG44. D: N6-Ac Lys in CHOK1 GS and CHO DG44. E: NAc-Met in CHOK1 GS and CHO DG44. F. NAc-His in CHO DG44. G: NAc-Asn in CHO DG44. H: NAc-Glu in CHO DG44. I: NAc-Phe in CHO DG44.

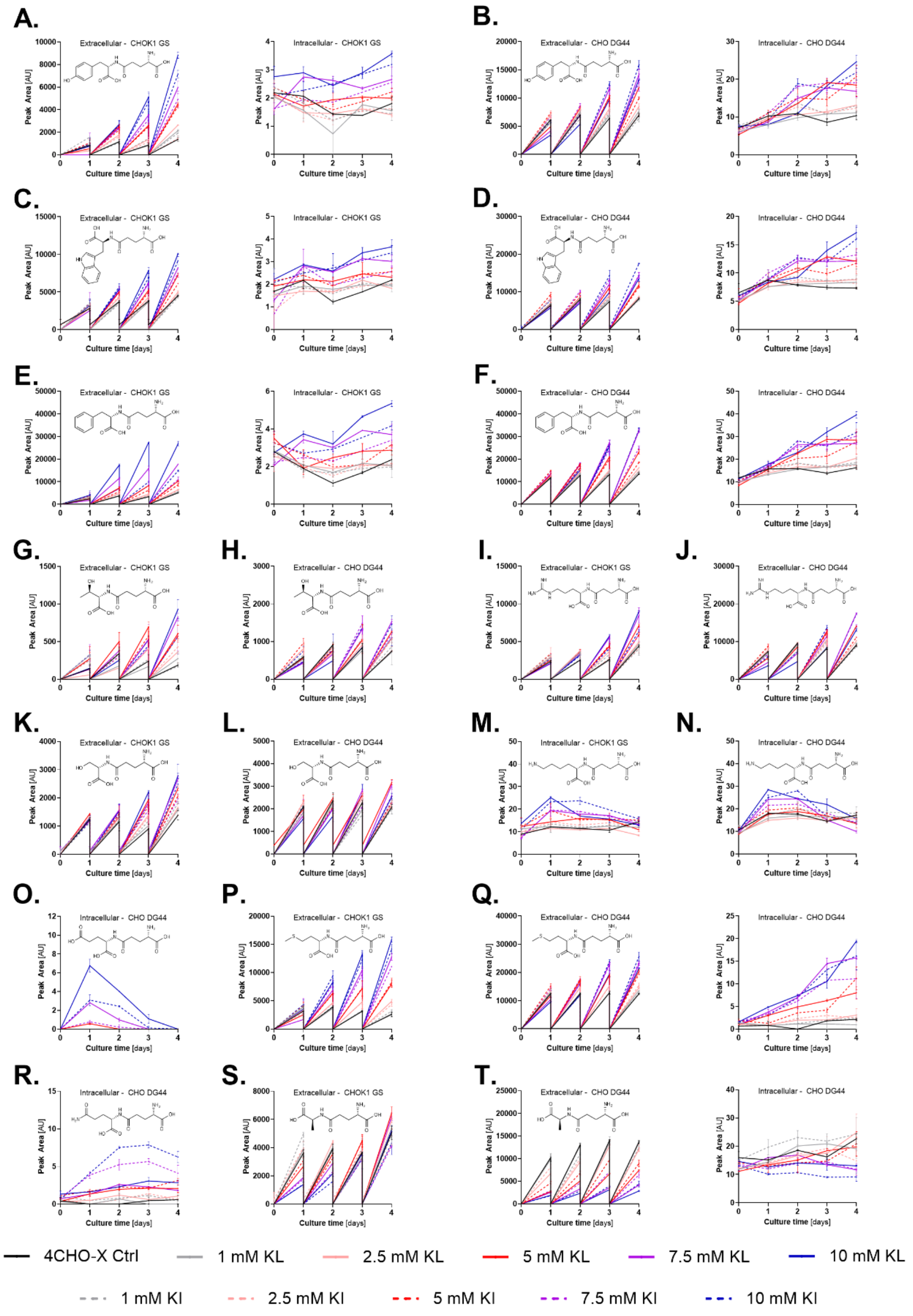

Supp. Figure 19: Abundance profiles of γ-glutamyl amino acids (γ-Glu-AA), identified in the spent media and intracellular content of CHO DG44 and CHOK1 GS during simulated perfusion. Intracellular values were normalized to the total protein content of the extracted pellet (n=2). A: γ-Glu-Tyr in CHOK1 GS. B: γ-Glu-Tyr in CHO DG44. C: γ-Glu-Trp in CHOK1 GS. D: γ-Glu-Trp in CHO DG44. E: γ-Glu-Phe in CHOK1 GS. F: γ-Glu-Phe in CHO DG44. G: γ-Glu-Thr in CHOK1 GS. H: γ-Glu-Thr in CHO DG44. I: γ-Glu-Arg in CHOK1 GS. J: γ-Glu-Arg in CHO DG44. K: γ-Glu-Ser in CHOK1 GS. L: γ-Glu-Ser in CHO DG44. M: γ-Glu-Lys in CHOK1 GS. N: γ-Glu-Lys in CHO DG44. O: γ-Glu-Glu in CHO DG44. P: γ-Glu-Met in CHOK1 GS. Q: γ-Glu-Met in CHO DG44. R: γ-Glu-Gln in CHO DG44. S: γ-Glu-Ala in CHOK1 GS. T: γ-Glu-Ala in CHO DG44.

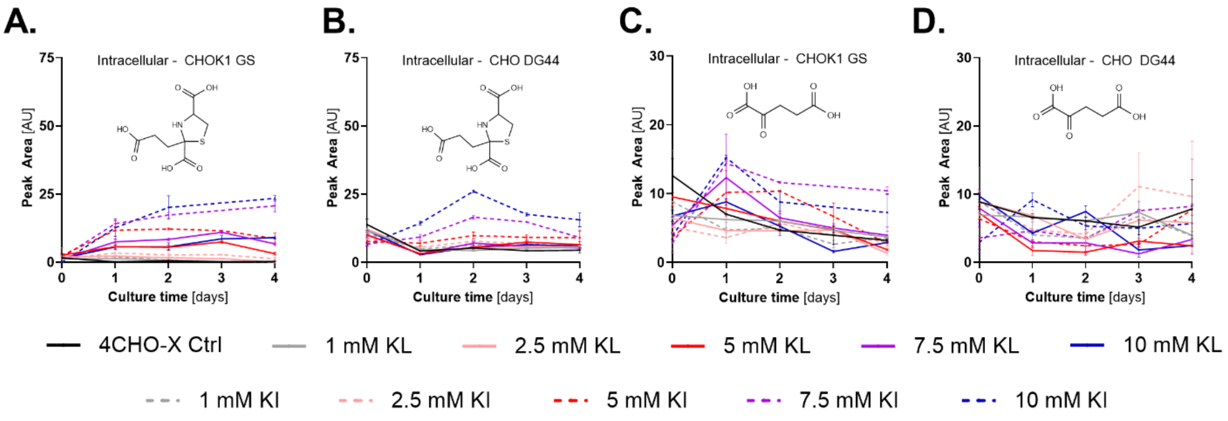

Supp. Figure 20: Abundance profiles of ketoglutarate (KG) and the thiazolidine CKG, formed from cysteine and KG, which was identified in the intracellular content of CHO DG44 and CHOK1 GS during a simulated perfusion process. Intracellular values were normalized to the total protein content of the extracted pellet (n=2). A: CKG with CHOK1 GS. B: CKG with CHO DG44. C: KG with CHOK1 GS. D: KG with CHO DG44.
